# Immunological Changes in Blood of Newborns Exposed to Anti-TNF-α during Pregnancy

**DOI:** 10.3389/fimmu.2017.01123

**Published:** 2017-09-21

**Authors:** Ana Esteve-Solé, Àngela Deyà-Martínez, Irene Teixidó, Elena Ricart, Macarena Gompertz, Maria Torradeflot, Noemí de Moner, Europa Azucena Gonzalez, Ana Maria Plaza-Martin, Jordi Yagüe, Manel Juan, Laia Alsina

**Affiliations:** ^1^Allergy and Clinical Immunology Department, Hospital Sant Joan de Déu, Institut de Recerca Pediàtrica Hospital Sant Joan de Déu, Esplugues de Llobregat, Spain; ^2^Functional Unit of Clinical Immunology, Sant Joan de Déu-Hospital Clinic, Barcelona, Spain; ^3^BCNatal – Barcelona Center of Maternal-Fetal and Neonatal Medicine, Hospital Clínic de Barcelona, Barcelona, Spain; ^4^Gastroenterology Department, Centro de Investigación Biomédica en Red de Enfermedades Hepáticas y Digestivas (CIBEREHD), IDIBAPS, Barcelona, Spain; ^5^Department of Gastroenterology, Hospital Clínic de Barcelona, IDIBAPS, University of Barcelona, Barcelona, Spain; ^6^Immunology Department, Centre de Diagnòstic Biomèdic, Hospital Clínic de Barcelona, Universitat de Barcelona, IDIBAPS, Barcelona, Spain

**Keywords:** adalimumab, inflammatory bowel disease, infliximab, monoclonal antibodies, pregnancy, prenatal exposure

## Abstract

**Background:**

Although anti-TNF-α monoclonal antibodies are considered safe during pregnancy, there are no studies on the development of the exposed-infant immune system. The objective was to study for the first time the impact of throughout pregnancy exposure to anti-TNF-α has an impact in the development of the infant’s immune system, especially B cells and the IL-12/IFN-γ pathway.

**Methods:**

Prospective study of infants born to mothers with inflammatory bowel disease treated throughout pregnancy with anti-TNF-α (adalimumab/infliximab). Infants were monitored both clinically and immunologically at birth and at 3, 6, 12, and 18 months.

**Results:**

We included seven patients and eight healthy controls. Exposed infants had detectable levels of anti-TNF-α until 6 months of age; they presented a more immature B- and helper T-phenotype that normalized within 12 months, with normal immunoglobulin production and vaccine responses. A decreased Treg cell frequency at birth that inversely correlated with mother’s peripartum anti-TNF-α levels was observed. Also, a decreased response after mycobacterial challenge was noted. Clinically, no serious infections occurred during follow-up. Four of seven had atopia.

**Conclusion:**

This study reveals changes in the immune system of infants exposed during pregnancy to anti-TNF-α. We hypothesize that a Treg decrease might facilitate hypersensitivity and that defects in IL-12/IFN-γ pathway might place the infant at risk of intracellular infections. Pediatricians should be aware of these changes. Although new studies are needed to confirm these results, our findings are especially relevant in view of a likely increase in the use of these drugs during pregnancy in the coming years.

## Introduction

Anti-TNF-α monoclonal antibodies (mAb) have revolutionized inflammatory bowel disease (IBD) treatment (1–4). IBD onset is frequently observed in women during childbearing age (3, 4); nevertheless, pregnancy is not associated with IBD improvement (5–9). Active IBD can lead to increased pre-term deliveries and spontaneous abortion, and sustained remission of IBD is often only achievable with anti-TNF-α treatment. Anti-TNF-α mAb cross placenta mostly from week 28 of gestation to delivery (10); they are included in category B (no apparent risk) of FDA-classification for pregnancy risk. However, drug level’s safety in newborns and the full consequences of such exposure in newborn’s immune system development are unclear.

Several recent studies in extensive cohorts [PIANO (*n* = 426), OTIS registry (*n* = 74), and CRIB study (*n* = 31)] of infants exposed to anti-TNF-α drugs during, at least, the second and third trimester of pregnancy showed that both anti-TNF-α adalimumab (ADA) and infliximab (IFX) are detectable in infants from treated mothers for 12 months after birth while certolizumab was barely detectable. Infants showed no apparent major clinical consequences (11–14). Nevertheless, a fatal case of disseminated Bacillus Calmette–Guérin (BCG) disease after vaccination was reported in an infant whose mother had been treated with high doses of IFX during pregnancy (15). It is recommended to delay immunization with live vaccines until 12 months or after confirmation of negative drug levels (13, 16).

It has been hypothesized that biologic drugs targeting immune-system molecules can lead to phenotypes resembling primary immune deficiencies (PID) related to the inhibited or modulated pathway (17). Although there are not known PID caused specifically by a deficiency in TNF-α, it plays a key role in the IL-12/IFN-γ pathway, responsible for the response to intracellular pathogens. Infections observed in adult patients treated with anti-TNF-α mAbs resemble those observed in patients with Mendelian Susceptibility to Mycobacterial Disease (OMIM 209950) (18), caused by monogenic errors in the IL-12/IFN-γ pathway. Severe or recurrent infections by atypical/non-pathogenic mycobacteria and other intra-macrophagic infections, including salmonellosis are characteristic in patients with Mendelian Susceptibility to Mycobacterial Disease and in patients treated with anti-TNF-α (19). Anti-TNF-α treatment alters the IL-12/IFN-γ pathway by inhibiting IL-12p70 production in response to CD40L stimulation (18). Besides, it is thought that it plays a pivotal role in B cell development: TNF-α knockout mice present abnormal B cell structures. These alterations lead to a decreased humoral responses and increased infection risk (20).

The in-depth study of the effect of prenatal anti-TNF-α exposure on a developing immune system remains a relevant clinical question. Therefore, this was the aim of our study: our hypothesis was that throughout exposure during pregnancy to anti-TNF-α affects the development of the infant’s immune system, especially B-cells (20, 21) and the IL-12/IFN-γ pathway (18). To our knowledge, this is the first study to shed light on how prenatal anti-TNF-α influences the human immune system development.

## Materials and Methods

### Patients Included

We conducted a prospective study (January 2014–January 2016) of infants born to mothers with IBD who received anti-TNF-α mAb (ADA or IFX) throughout during pregnancy, including the third trimester. Infants undergoing immunosuppressive treatment or with an immunodeficiency were excluded. All IBD patients included in the study were recruited from the IBD Unit of Hospital Clinic de Barcelona (HCB), and underwent close monitoring of pregnancy and childbirth. At the time of the initiation of our study, the IBD Unit of HCB was the only institution in our region maintaining this treatment during throughout pregnancy and all patients but one fulfilling inclusion criteria were enrolled in the study.

This study was carried out in accordance with the recommendations of Ley General de Sanidad (25/4/1986) Art. 10, with written informed consent from all subjects. The protocol was approved by the ethics committee of the Hospital Sant Joan de Déu (Comité Ético de Investigaciones Clínicas number PIC-50-12). All patients included in the study signed the informed consent, complying with current legislation.

We included seven exposed-infants and eight healthy controls. Exposed-infants were monitored both clinically and immunologically at birth and at 3, 6, 12, and 18 months. On the delivery day, 20 ml of heparinized blood was extracted from umbilical cord blood (seven exposed-infants and eight controls). From 3 months, 6 ml heparinized blood and 2.5 ml sera without anticoagulant factor were obtained from all exposed-infants but 2, due to the mother’s refusing subsequent blood draws after an unsuccessful first attempt.

### Immune and Clinical Follow-up

Immune parameter study included: quantification of anti-TNF-α blood levels, basic T/B/NK immunophenotype, T-cell and B-cell subphenotypes with evaluation of regulatory cells (Treg and Breg), lymphocyte proliferation to mitogens, and IFN-γ/IL-12 pathway (Tables S1 and S2 in Supplementary Material). Common procedures as immunophenotyping and cell proliferation are detailed in the supplementary methods. To facilitate reading and interpretation, populations appear in the text with a given name, while markers definitions are presented in Table 1.

**Table 1:** Lymphocyte subpopulation cell markers.

Name	Cell marker
T lymphocytes	CD45^+^CD3^+^CD19^−^
B lymphocytes	CD45^+^CD3^−^CD19^+^
NK cells	CD45^+^CD3^−^CD16CD56^+^
CD4 T cells	CD45^+^CD3^+^CD4^+^
CD8 T cells	CD45^+^CD3^+^CD8^+^
Double-negative T cells	CD45^+^CD3^+^CD4^−^CD8^−^
TCRγδ T cells	CD3^+^TCRγδ^+^
TCRαβ T cells	CD3^+^TCRαβ^+^
Naive T cells	CD3^+^CD45RA^+^CD45RO^−^
Cytotoxic naive T cells	CD3^+^CD45RA^+^CD45RO^−^CD8^+^
Helper naive T cells	CD3^+^CD45RA^+^CD45RO^−^CD8^−^
Memory T cells	CD3^+^CD45RO^+^
Cytotoxic memory T cells	CD3^+^CD45RO^+^CD8^+^
Helper memory T cells	CD3^+^CD45RO^+^CD8^−^
Late-memory T cells	CD3^+^CD45RA^−^CD45RO^+^
Cytotoxic late-memory T cells	CD3^+^CD45RA^−^CD45RO^+^CD8^+^
Helper late-memory T cells	CD3^+^CD45RA^−^CD45RO^+^CD8^−^
Early-memory T cells	CD3^+^CD45RA^+^CD45RO^+^
Cytotoxic early-memory T cells	CD3^+^CD45RA^+^CD45RO^+^CD8^+^
Helper early-memory T cells	CD3^+^CD45RA^+^CD45RO^+^CD8^−^
T regulatory cells	CD45^+^CD3^+^CD4^+^CD25^hi^CD127^low^FoxP3^+^
IgD^+^IgM^+^ B cells	CD19^+^IgD^+^IgM^+^
Marginal zone B cells	CD19^+^IgD^+^IgM^+^CD27^+^
Naive B cells	CD19^+^IgD^+^CD27^−^
Transitional B cells	CD19^+^IgM^+^CD38^hi^
B regulatory cells	CD19^+^CD24^hi^CD38^hi^
IgD^+^IgM^−^ B cells	CD19^+^IgD^+^IgM^−^
Switched B cells	CD19^+^IgD^−^IgM^−^
Switched memory B cells	CD19^+^IgD^−^IgM^−^CD27^+^
IgM only B cells	CD19^+^IgD^−^IgM^+^
Activated B cells	CD19^+^CD38^low^CD21^low^
Plasmablasts	CD19^+^IgM^low^CD38^hi^

#### Study of IL-12/IFN-γ Pathway in Response to Mycobacterial Stimulus

For the study of IL-12/IFN-γ pathway, we performed a whole blood culture (22). Heparinized blood was diluted 1:2 in complete medium [RPMI (Gibco, Grand Island, New York, NY, USA)] supplemented with 10% heat-inactivated fetal calf serum (FCS; Sigma-Aldrich, St. Louis, MO, USA), 1 µg/ml penicillin, and 1 µg/ml streptomycin (Invitrogen, Grand Island, New York, NY, USA) and incubated at 37°C in a 5% CO_2_ humidified incubator for 48 h. To assess the effect of anti-TNF-α mAb on the function of IL-12/IFN-γ pathway, the same whole blood culture was performed in two ways: using blood from exposed infants without washing and using the blood washed with PBS three times to eliminate anti-TNF-α drug. After the washing, complete medium (RPMI with 10% FBS and penicillin/streptomycin) was added to restore blood to the same volume as before the washing with PBS. In this way, we maintained cellular amounts of cells and cell concentration in the two conditions assayed. Activation conditions: medium alone, live BCG (*M. bovis* BCG, Pasteur substrain) at a multiplicity of infection of 20 BCG per leukocyte, BCG plus human recombinant IL-12p70 (hrIL-12p70, 20 ng/ml, Miltenyi Biotec, Germany), BCG plus hrIFN-γ (5,000 IU/ml; Imukin, Boehringer Ingelheim, Germany) as described elsewhere (22). We analyzed activation markers after 48 h of culture by flow cytometry.

Cytokine production determination was assessed by Luminex (Millipore, Billerica, MA, USA) at 48-h culture point following the manufacturer’s instructions. Briefly, supernatants were incubated for 2 h with corresponding anti-cytokine magnetic beads, and then washed with 1× washing buffer and stained with detection antibodies (provided) for 1 h. Strepatividin-PE was then added for 30 more minutes. During all incubation steps, the plate was agitated at 650 rpm. After washing, plate was agitated for 15 min at 650 rpm and read in the xMAP Luminex reader (Waltham, MA, USA). IL-17 detection was assessed by ELISA (Invitrogen, Carlsbad, CA, USA) at 48 h culture point following manufacturer’s instructions.

### Statistical Analysis

As data did not follow a Gaussian distribution, unpaired *t*-test was performed to compare different cell populations between exposed and non-exposed infants. Significance of correlation between populations/drug levels was studied with Spearman test for non-parametric populations.

Statistical significance of functional studies was performed with two-way ANOVA test and Bonferroni post-test. For the comparison between the results obtained with autologous sera and washed condition, two-way ANOVA for repeated measures was used. In heat-map representation, blue corresponds with the minimum, red with the maximum, and yellow with one for each parameter.

Statistical analysis and graphical representation of the data were performed with Prism5 software (GraphPad, La Jolla, CA, USA) and Microsoft Excel (2010). Detailed results (mean ± SE of the mean and *p*) for immune phenotype are detailed in Tables 2 and 3.

**Table 2:** Leukocyte populations in umbilical cord blood of exposed and non-exposed infants.

		Exposed (*n* =6)		Non-exp (*n* = 8)		*p*	ADA-exposed (*n* = 4)			IFX-exposed (*n* = 2)		
		Mean	SEM	Mean	SEM		Mean	SEM	*p*	Mean	SEM	*p*
Absolute numbers (10^3^ cells/μl blood)	Leukocytes	16.06	3.12	13.12	1.00	0.28	20.15	3.65	0.15	12.36	6.15	>0.9999
	Lymphocytes	7.18	1.47	5.39	0.41	0.08	9.31	1.47	0.02	5.27	2.93	>0.9999
	Neutrophils	7.29	1.83	6.18	0.74	0.85	8.54	2.81	0.9	6.48	3.42	0.84
	Monocytes	1.04	0.24	1.16	0.10	0.94	1.28	0.26	0.15	0.44	0.04	0.04
	Basophils	0.11	0.02	0.39	0.11	0.09	0.09	0.03	0.07	NA	NA	NA
	Eosinophils	0.44	0.21	0.08	0.02	0.07	0.63	0.55	0.004	NA	NA	NA

Absolute numbers (cells/μl blood)	T lymphocytes	5593.00	1006.00	3479.00	256.70	0.04*	6298	1045	0.004*	4184	2376	1
	B lymphocytes	959.20	303.80	978.60	166.30	0.85	1112	420.4	0.57	653.6	428.8	0.71
	NK cells	1100.00	304.00	796.40	217.80	0.66	1552	164.8	0.11	196.8	4.92	0.18
	CD4 T cells	3935.00	779.40	2442.00	169.80	0.14	4470	854.2	0.49	2864	1736	1
	CD8 T cells	1523.00	294.70	925.10	101.00	0.14	1736	343.5	0.07	1097	567.9	0.89
	Double-negative T cells	182.80	45.22	70.90	10.66	0.01*	217.6	59.69	0.008*	113.4	45.37	0.09

% of T cells	TCRγδ T cells	3.57	0.79	2.11	0.28	0.28	3.12	0.67	0.26	2.39	0.38	0.67
	TCRαβ T cells	94.84	0.80	94.26	0.45	0.15	95.18	0.78	0.57	96.15	0.25	0.07
	Naive T cells	62.72	6.82	46.90	2.32	0.13	54.88	7.226	0.47	78.4	3.9	0.07
	Cytotoxic naive T cells	15.11	1.22	16.20	1.24	0.82	13.25	0.4575	0.25	18.82	0.625	0.28
	Helper naive T cells	47.62	6.08	30.73	2.33	0.13	41.64	7.293	0.47	59.59	4.525	0.07
	Memory T cells	34.68	7.98	52.65	2.30	0.13	44.9	7.391	0.48	14.25	0.35	0.07
	Cytotoxic memory T cells	11.74	4.11	9.77	1.17	0.7	11.74	4.108	0.61	5.675	0.305	0.07
	Helper memory T cells	22.95	5.393	42.9	1.784	0.009*	22.95	5.393	0.04*	8.575	0.655	0.07
	Late-memory T cells	9.07	1.39	8.05	0.80	0.81	8.948	2.156	0.91	9.31	0.89	0.43
	Cytotoxic late-memory T cells	2.55	0.70	0.12	0.03	0.002*	2.21	1.046	0.01*	3.225	0.145	0.07
	Helper late-memory T cells	6.52	1.05	7.92	0.78	0.13	6.738	1.625	0.25	6.085	0.745	0.28
	Early-memory T cells	25.64	8.53	44.87	2.09	0.18	35.73	8.952	0.61	5.46	1.02	0.07
	Cytotoxic early-memory T cells	9.22	3.85	9.69	1.15	0.69	12.47	5.132	0.61	2.715	0.705	0.07
	Helper early memory T cells	16.42	5.71	35.18	1.90	0.03*	23.26	5.901	0.11	2.745	0.315	0.07

% of CD4 T cells	T regulatory cells	0.24	0.04	0.5	0.10	0.04	0.22	0.037	0.049	0.29	0.11	0.4

% of B cells	IgD^+^IgM^+^ B cells	91.97	2.38	92.03	1.30	0.46	90.13	3.20	0.920	95.65	1.85	0.29
	Marginal zone B cells	20.16	9.02	40.74	2.46	0.03*	29.58	40.74	0.190	1.33	0.45	0.04*
	Naive B cells	72.21	10.87	47.70	3.49	0.045*	60.77	12.82	0.270	95.11	1.42	0.04*
	Transitional B cells	59.75	10.78	53.89	4.88	0.44	50.43	13.66	0.920	78.40	10.00	0.09
	B regulatory cells	49.31	1.42	34.39	2.49	0.0007*	47.62	1.31	0.004*	53.55	0.45	0.04*
	IgD^+^IgM^−^ B cells	3.36	2.13	3.64	1.65	0.72	4.35	3.18	0.780	1.37	1.15	0.89
	Switched B cells	2.84	1.00	3.90	1.30	0.045*	3.59	1.35	0.130	1.32	0.79	0.17
	Switched memory B cells	2.26	0.82	2.57	0.58	0.35	2.76	1.16	0.630	1.28	0.78	0.4
	IgM only B cells	1.81	0.16	0.43	0.09	0.002*	1.90	0.24	0.010*	1.64	0.10	0.04*
	Activated B cells	1.23	0.73	0.05	0.03	0.003*	1.74	1.03	0.010*	0.21	0.12	0.09
	*Plasmablasts*	1.33	0.32	1.39	0.28	0.94	1.81	0.16	0.500	0.7	0.41	0.27

*ADA, adalimumab; IFX, infliximab*.

*indicates statistically significant p-values.

**Table 3:** Activation markers’ expression and cytokine production after 48 h whole blood culture with different stimulations.

		Ratio stim/basal				Ratio w_exp/exp			
Variable		Stimulus	Non-exp	Exp	w_exp	UCB	3 months	6 months	12 months
**ACTIVATION MARKERS EXPRESSION**									
CD71	Freq%	Bacillus Calmette–Guérin (BCG)	2.36	1.416	1.162	2.307	3.434	4.004	3.788
		BCG IL-12	2.379	1.768	1.434	2.227	2.768	3.826	3.368
		BCG IFN-γ	3.018	1.682	1.252	1.883	3.204	4.394	3.51

			**Non-exp vs exp**	**Non-exp vs w_exp**	**Exp vs w_exp**	***p* of the effect of time in the changes observed**			

		*p*	0.06	0.06	0.17	0.13			
	MFI	BCG	1.154	1.238	1.116	3.443	2.164	2.016	1.93
		BCG IL-12	1.183	1.168	1.218	3.487	2.034	1.926	1.75
		BCG IFN-γ	1.266	1.28	1.032	2.733	2.006	2.026	1.69

			**Non-exp vs exp**	**Non-exp vs w_exp**	**Exp vs w_exp**	***p* of the effect of time in the changes observed**			

		*p*	0.91	0.71	0.27	0.003			
CD69	Freq%	BCG	2.306	2.052	1.904	1.14	1.434	1.018	2.11
		BCG IL-12	4.359	2.664	3.778	1.103	1.412	1.09	2.143
		BCG IFN-γ	4.829	2.69	3.424	2.653	1.48	1.15	1.938

			**Non-exp vs exp**	**Non-exp vs w_exp**	**Exp vs w_exp**	***p* of the effect of time in the changes observed**			

		*p*	0.13	0.13	0.09	0.15			
	MFI	BCG	5.478	2.106	1.838	1.513	1.526	0.918	1.02
		BCG IL-12	11.34	2.57	4.38	1.647	1.674	1.044	1.438
		BCG IFN-γ	12.22	2.464	3.232	1.887	1.66	1.038	1.008

			**Non-exp vs exp**	**Non-exp vs w_exp**	**Exp vs w_exp**	***p* of the effect of time in the changes observed**			

		p	0.001*	0.004*	0.02*	0.009*			
HLA-DR	Freq%	BCG	1.123	1.854	1.674	1.053	1.08	0.938	0.9725
		BCG IL-12	1.231	2.18	1.854	1.03	1.038	0.94	1.018
		BCG IFN-γ	1.443	2.016	1.61	0.9867	1.03	0.976	0.955

			**Non-exp vs exp**	**Non-exp vs w_exp**	**Exp vs w_exp**	***p* of the effect of time in the changes observed**			

		p	0.21	0.1	0.26	0.58			
	MFI	BCG	2.024	1.166	1.926	2.673	1.368	0.962	1.318
		BCG IL-12	2.38	1.19	2.198	2.093	1.164	1.046	1.165
		BCG IFN-γ	2.564	1.482	2.404	2.99	1.338	1.204	1.213

			**Non-exp vs exp**	**Non-exp vs w_exp**	**Exp vs w_exp**	***p* of the effect of time in the changes observed**			

		p	0.003*	0.05	0.1	0.009*			

**CYTOKINE SECRETION**									

IFN-γ	BCG	282.1	12.68	85.44	15.97	3.378	0.946	1.195	
	BCG IL-12	2994	2835	6680	10.4	1.616	7.866	0.9375	

		**Non-exp vs exp**	**Non-exp vs w_exp**	**Exp vs w_exp**	***p* of the effect of time in the changes observed**				

	*p*	0.87	0.22	0.18	0.13				
IL-10	BCG	775.6	510	611.5	0.4733	0.376	0.442	0.5175	
	BCG IL-12	841.5	447.4	523.9	0.4567	0.202	0.366	0.35	
	BCG IFN-γ	120.6	60.8	22.45	0.6933	0.304	0.302	0.295	

		**Non-exp vs exp**	**Non-exp vs w_exp**	**Exp vs w_exp**	***p* of the effect of time in the changes observed**				

	*p*	0.42	0.5	0.74	0.61				
IL-12p70	BCG	0.3638	2.132	0.032	0.54	0.2	0.2	0.25	
	BCG IFN-γ	38.23	73.18	88.62	4.78	2.87	1.308	3.07	

		**Non-exp vs exp**	**Non-exp vs w_exp**	**Exp vs w_exp**	***p* of the effect of time in the changes observed**				

	*p*	0.48	0.44	0.5	0.5				
IL1-RA	BCG	42.72	144.4	69.55	1.157	1.272	1.69	1.83	
	BCG IL-12	39.82	140.4	69.27	1.197	1.07	1.45	1.385	
	BCG IFN-γ	47.04	144.8	70.63	0.93	1.086	0.968	1.435	

		**Non-exp vs exp**	**Non-exp vs w_exp**	**Exp vs w_exp**	***p* of the effect of time in the changes observed**				

	*p*	0.07	0.32	0.07	0.12				
IL-1β	BCG	4223	2176	2069	0.94	0.626	1.284	0.6775	
	BCG IL-12	4361	2232	2099	1.063	0.838	1.76	0.7725	
	BCG IFN-γ	5264	2180	2139	0.8967	0.738	0.948	0.5525	

		**Non-exp vs exp**	**Non-exp vs w_exp**	**Exp vs w_exp**	***p* of the effect of time in the changes observed**				

	*p*	0.06	0.06	0.39	0.05				
IL-6	BCG	953.3	2676	3551	0.7233	4.172	0.882	0.785	
	BCG IL-12	1783	2703	3538	0.61	2.124	1.114	0.9425	
	BCG IFN-γ	1002	3176	3571	5.92	0.704	0.614	0.6575	

		**Non-exp vs exp**	**Non-exp vs w_exp**	**Exp vs w_exp**	***p* of the effect of time in the changes observed**				

	*p*	0.06	0.03*	0.52	0.25				
TNF-α	BCG	621.8	42.86	243.7	20.91	18.15	6.604	1.06	
	BCG IL-12	638	44.09	165.8	24.34	51.46	9.276	1.23	
	BCG IFN-γ	818.8	87.72	509.1	34.44	40.06	3.528	0.85	

		**Non-exp vs exp**	**Non-exp vs w_exp**	**Exp vs w_exp**	***p* of the effect of time in the changes observed**				

	*p*	0.0002*	0.02*	0.005	0.02				

*Stim, stimulation; non-exp, healthy neonate; exp, infants exposed to anti-TNF-α during pregnancy, blood cultured with autologous sera; w_exp, infants exposed to anti-TNF-α during pregnancy, blood cultured after PBS washing; MFI, mean fluorescence intensity; freq, frequency of lymphocytes*.

*Age expressed in months*.

*indicates statistically significant p-values.

## Results

### All Studied Mothers Presented Therapeutic Levels of Anti-TNF-α

Seven moderate-to-severe IBD pregnant patients (mean 34 years old, range 27–36) with long-lasting IBD (mean 9 years since diagnosis, range 2.5–11) were treated with anti-TNF-α for a period longer than 6-months prior to pregnancy. Six patients suffered from Crohn’s disease (CD) and one from extended ulcerative colitis. Five patients were treated with ADA, and two with IFX. All mothers presented supra-therapeutic levels of anti-TNF-α (ADA >4 μg/ml; IFX >3 μg/ml) before or during pregnancy. Three patients were treated with other immunosuppressive drugs—2 with azathioprine (AZA) and one with prednisolone—and two of them had active IBD during pregnancy (Table S3 in Supplementary Material).

For all patients, the interval between the last dose of drug and the delivery was ≤7 days (range: 3–7 days). The delivery was programmed only in two patients; the five remaining mothers gave birth by caesarian section (1) or vaginal (4) delivery, with one preterm infant (35 weeks in one mother with active disease); while the remaining 4-cases delivered at 39 weeks on average (range 37–41). All newborns were normal weight for gestational age, Apgar 9/10, and did not present any malformations. Five mothers breastfed.

### Levels of Anti-TNF-α in the Infant

**Figure 1:**
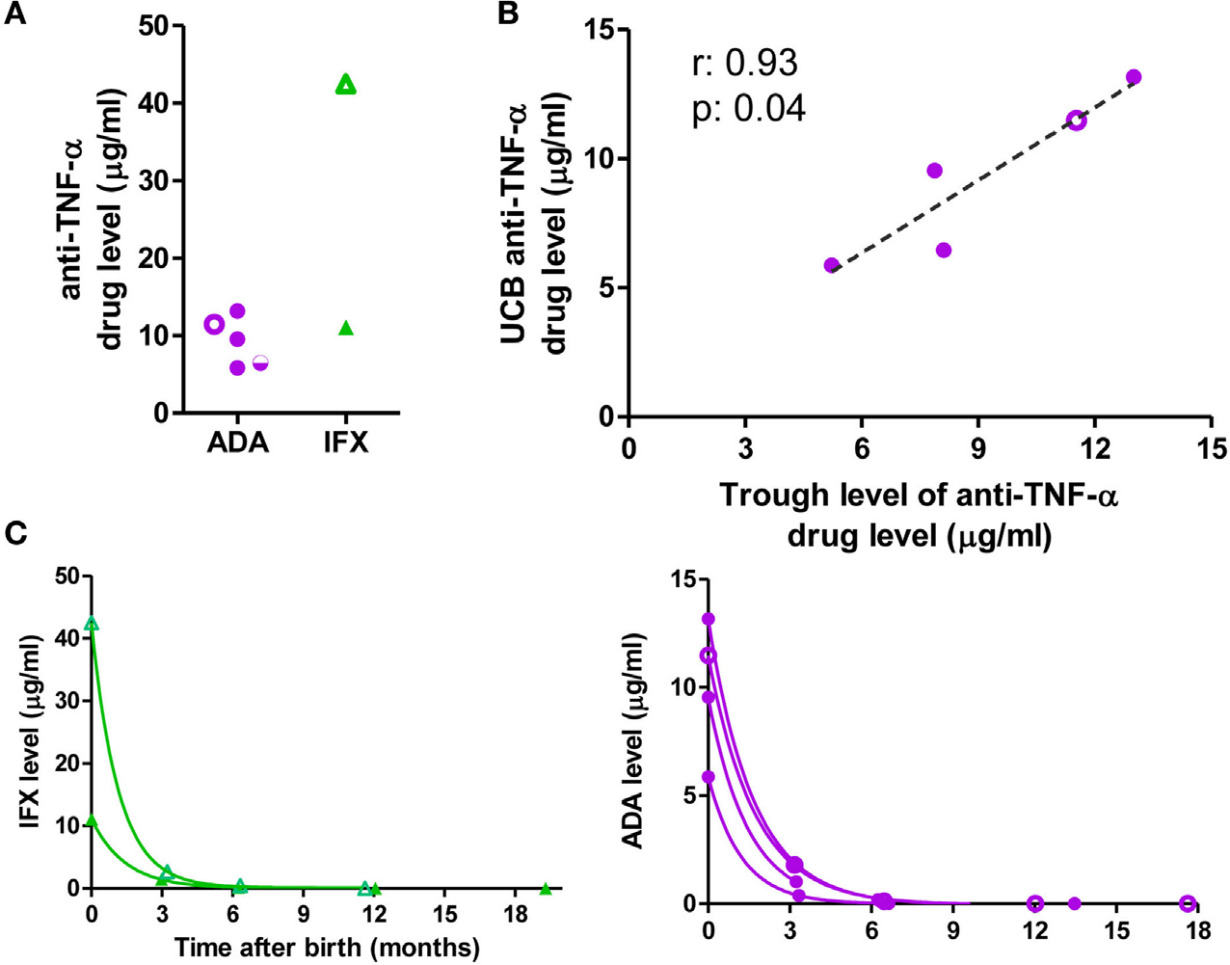
Anti-TNF-α drug levels. **(A)** Anti-TNF-α levels in cord blood. **(B)** Correlation between mother’s adalimumab (ADA) trough level during the third trimester of pregnancy and level in cord blood. **(C)** Clearance of the anti-TNF-α monoclonal antibodies. Purple circles: ADA-exposed; green triangles: infliximab (IFX)-exposed, empty symbols: co-exposure with azathioprine; half-empty symbols: co-exposure with prednisone. ADA-exposed infants, *n* = 5; IFX-exposed infants, *n* = 2.

Exposed-infants had positive anti-TNF-α drug levels in cord blood (mean 11.42 µg/ml, range: 5.87–42.52 µg/ml; Figure 1A); with especially high levels in one IFX-exposed infant whose mother received the drug 4 days before delivery. There was a direct correlation between the trough level of ADA in the mother and the cord blood level (*r* = 0.9 *p* = 0.04); this correlation was not determinable in IFX patients due to the low number of samples (Figure 1B). The ratio between the mother and cord blood drug’s trough level was close to 1 (mean: 0.99) for ADA and higher for IFX (mean: 3.25).

Drug level clearance in the infant followed a one-phase decay with a mean half-life of 29.6 days (range 23.93–35.53 days), slightly longer than the described half-life of immunoglobulin (Ig) G (23). Drug levels were detectable until 6 months in all infants studied (Figure 1C; Table S4 in Supplementary Material).

### Mild Changes in Leukocyte and Lymphocyte Population in Exposed-Infants

There were no statistical differences in cord blood counts in red blood cells (Figure S1 in Supplementary Material), total leukocytes, lymphocytes, neutrophils, and basophils between exposed-infants and healthy controls (Figure S2 in Supplementary Material). However, although due to sample-size limitations comparisons between ADA- and IFX-exposed infants should be approached with caution. We have observed that, compared with healthy controls, IFX-exposed infants had lower monocyte count in cord blood (0.44 vs 1.16 × 10^3^ cells/μl blood) and that ADA-exposed infants presented increased eosinophil counts in cord blood (0.63 vs 0.08 × 10^3^ cells/μl blood; *p* = 0.004) (Figure S2 in Supplementary Material; Table 2).

Absolute number of T-cells was increased in cord blood of ADA-exposed-infants (6.294 vs 3.479 cells/μl of blood; *p* = 0.004), which fell within normal ranges at 12 months. No differences were observed in other lymphocyte subpopulations (Figures S2 and S3 in Supplementary Material). One ADA-treated patient, also treated with AZA, presented very low numbers of B-cells (0.54% of lymphocytes and 32 cells/μl of blood) (this effect was not observed in the other AZA-treated infant).

At 12 months of age, all exposed infants showed normal-for-age levels of leukocyte and lymphocyte subsets; neutrophils and NK were at the lower limit of the normal ranges (24) (Figure S2 in Supplementary Material; Table 2).

### Differences in T- and B-Lymphocyte Maturation Status in Exposed-Infants

**Figure 2:**
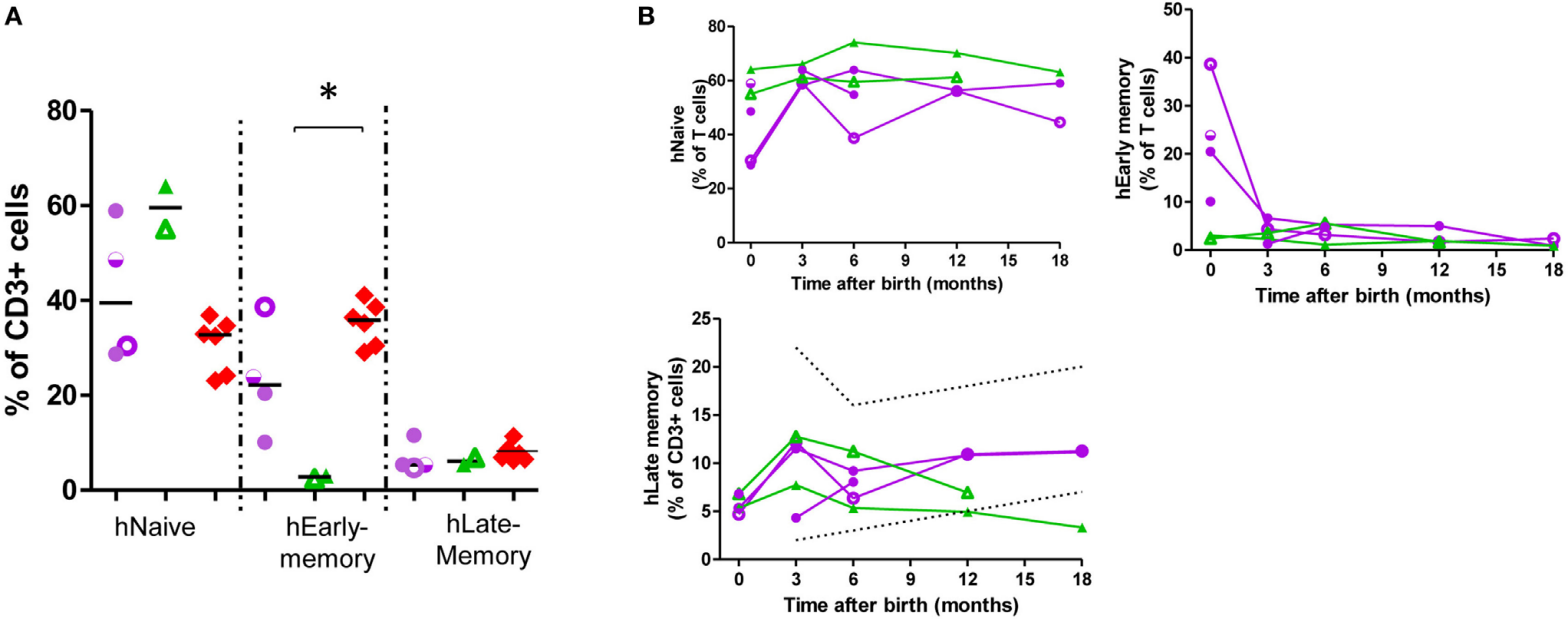
Helper CD45RO^+^CD45RA^+^ T-cells are at a low frequency in exposed infants cord blood. T-cell subpopulation frequency at birth **(A)** and at follow-up **(B)**. Dotted lines, described reference ranges; purple circles, adalimumab (ADA)-exposed; green triangles, infliximab (IFX)-exposed; red diamonds, cord blood healthy controls; empty symbols, azathioprine co-exposure; half-empty symbols, prednisone co-exposure. ADA-exposed infants, *n* = 4; IFX-exposed infants, *n* = 2.

In the T-cell compartment (Figure 2; Figure S4 in Supplementary Material; Table 2), there was a tendency toward an increase in the naïve population in exposed infants (62.72 vs 46.9% of T-cells, *p* = 0.13) due to T helper (Th)-subset. Furthermore, CD45RO^+^ (memory) population frequency was decreased among Th-cells (22.95 vs 42.9% of T-cells; *p* = 0.009) due to a decrease in Th-early-memory-cells (16.42 vs 35.18% of CD3^+^; *p* = 0.03) while there were no significant differences in late-memory T-cells. By contrast, there was an increase in the T cytotoxic Tc-memory-cells (2.55 vs 0.12% of T-cells; *p* = 0.002). At 12 months of age, CD45RO^+^ T-cells, both Th and Tc, were in range (25).

**Figure 3:**
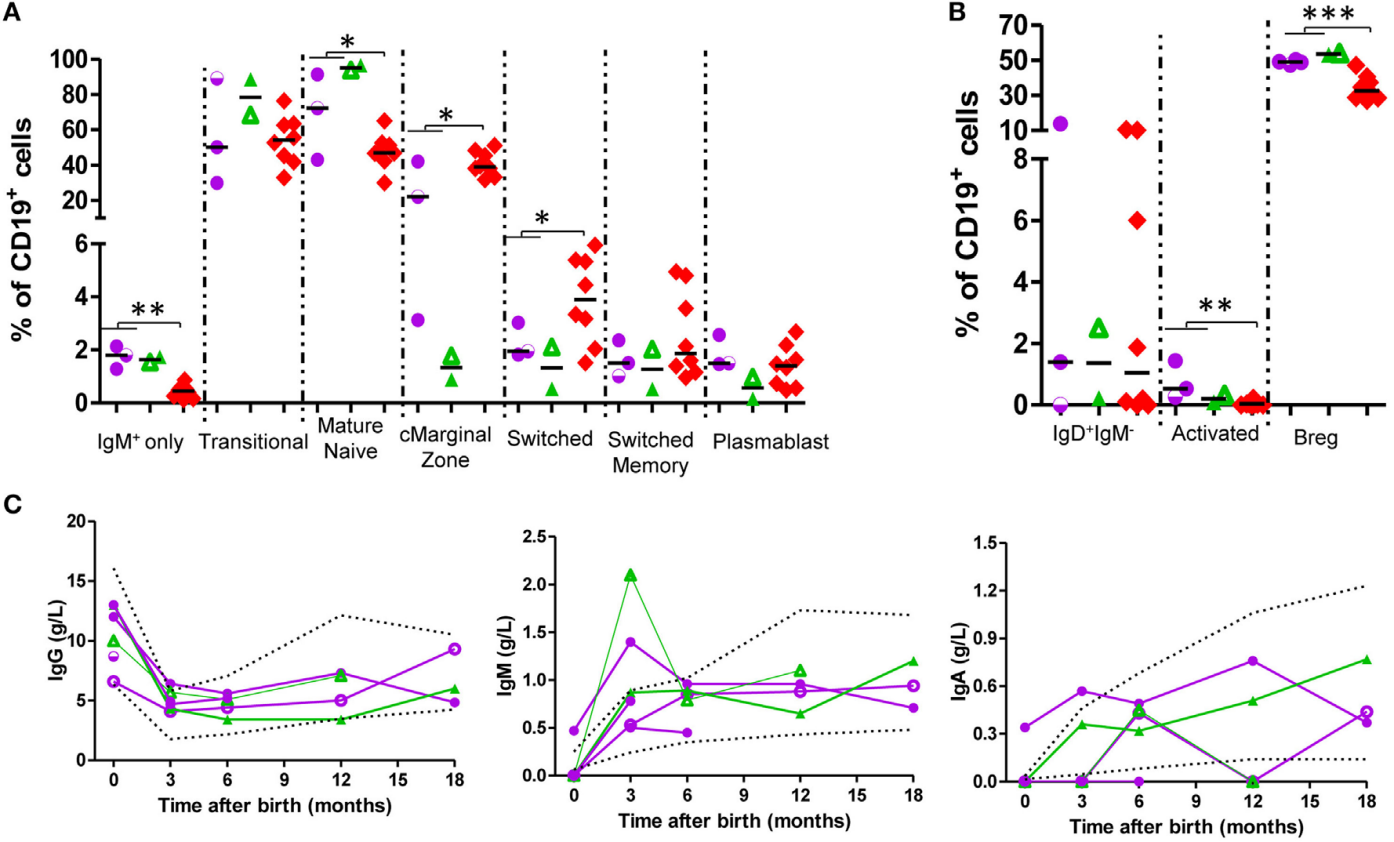
B-cells present higher levels of naïve markers and normal levels of immunoglobulins (Igs) in exposed infants. **(A,B)** B-cell subpopulation frequencies at birth. adalimumab (ADA)-exposed infants, *n* = 3; infliximab (IFX)-exposed infants, *n* = 2. **(C)** Ig production follow-up. ADA-exposed infants, *n* = 4; IFX-exposed infants, *n* = 2. Dotted lines, described reference ranges; purple circles, ADA-exposed; green triangles, IFX-exposed; red diamonds, cord blood healthy controls; empty symbols, azathioprine co-exposure; half-empty symbols: prednisone co-exposure.

In the B-cell compartment (Figure 3; Figure S5 in Supplementary Material; Table 2), naïve B-cell population frequency was greater in exposed infants (72.21 vs 47.70% of B cells, *p* = 0.045) and switched B-cell population was smaller (2.84 vs 3.90% of B-cells, *p* = 0.045). Circulating marginal zone B-cells were decreased (20.16 vs 40.74% of B cells; *p* = 0.03), and activated B-cells (1.23 vs 0.05% of B cells; *p* = 0.003) and IgM-only B-cell-frequency (1.81 vs 0.43% of B-cells, *p* = 0.002) were increased. Although at some time-points, some B-cell subsets showed values outside the range, at 12–18 months all values were within age-matched ranges (23, 24, 26, 27). In all exposed-infants, IgG, IgA, and IgM production were since birth in range; at 3 months there was an increase in IgM with respect to reference ranges (Figure 3C). IgG-responses to vaccines (tetanus, diphtheria, and pneumococcus) were normal (Table 3).

### Differences in Treg- and Breg Cells in Exposed Infants

**Figure 4:**
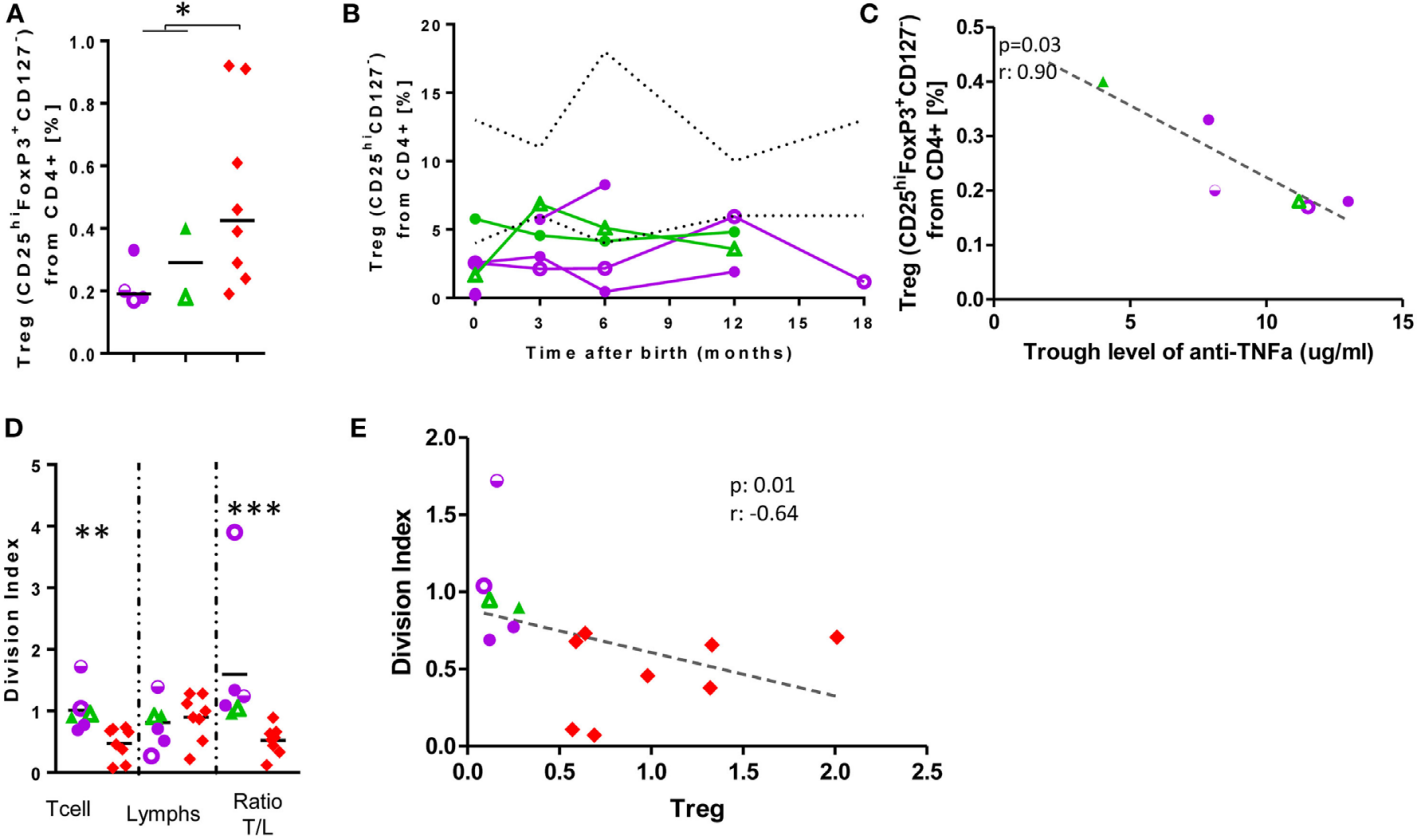
Low Treg levels at birth are not rescued and correlate with increased T-cell proliferation. Treg frequency at birth **(A)** and follow-up **(B)**. **(C)** Correlation between Treg frequency and mother’s anti-TNF-α trough level. **(D)** Division Index after 7-days’ stimulation with ConA. **(E)** Correlation between Treg frequency and T-cell division index. Dotted lines, described reference ranges; purple circles, adalimumab (ADA)-exposed; green triangles, infliximab (IFX)-exposed; red diamonds, cord blood healthy controls; empty symbols, azathioprine co-exposure; half-empty symbols: prednisone co-exposure. ADA-exposed infants, *n* = 4; IFX-exposed infants, *n* = 2.

In cord blood of anti-TNF-α-exposed-infants, Treg (Figure 4) were diminished (0.24 vs 0.5% of CD4^+^-cells; *p* = 0.04). An inverse correlation between mother’s anti-TNF-α trough level and Treg frequency was observed (*r* = −0.9; *p* = 0.03); at follow-up, almost all Treg values were below the lower reference limit (24). Treg frequency inversely correlated with T-cell proliferation to ConA (a weak T-cell mitogen; *r* = −0.64, *p* = 0.01; Figure 4). Ratio of division indexes between T-cells/total lymphocytes was significantly statistically higher in exposed infants than controls (1.6 vs 0.52; *p* = 0.01; Figure 4), while there were no differences when strong T-cell mitogens were used (Figure S6 in Supplementary Material). Inversely to the decrease in Treg, we observed in cord blood an increase in Breg (49.31 vs 34.39% of B-cells, *p* = 0.0007; Figure 4). We have observed similar amounts of IL-10 production in exposed-infant’s B cells as in cells from non-exposed infants. We also observed that Breg cell frequency positively correlated with the frequency of IL-10^+^ B cells (Figure S8 in Supplementary Material).

### Deficient Mycobactericidal Response in Exposed-Infants

We evaluated anti-mycobacterial response by studying surface activation markers and cytokine secretion after whole blood cultures in the presence (non-washed condition) and absence (washed condition) of autologous sera.

**Figure 5:**
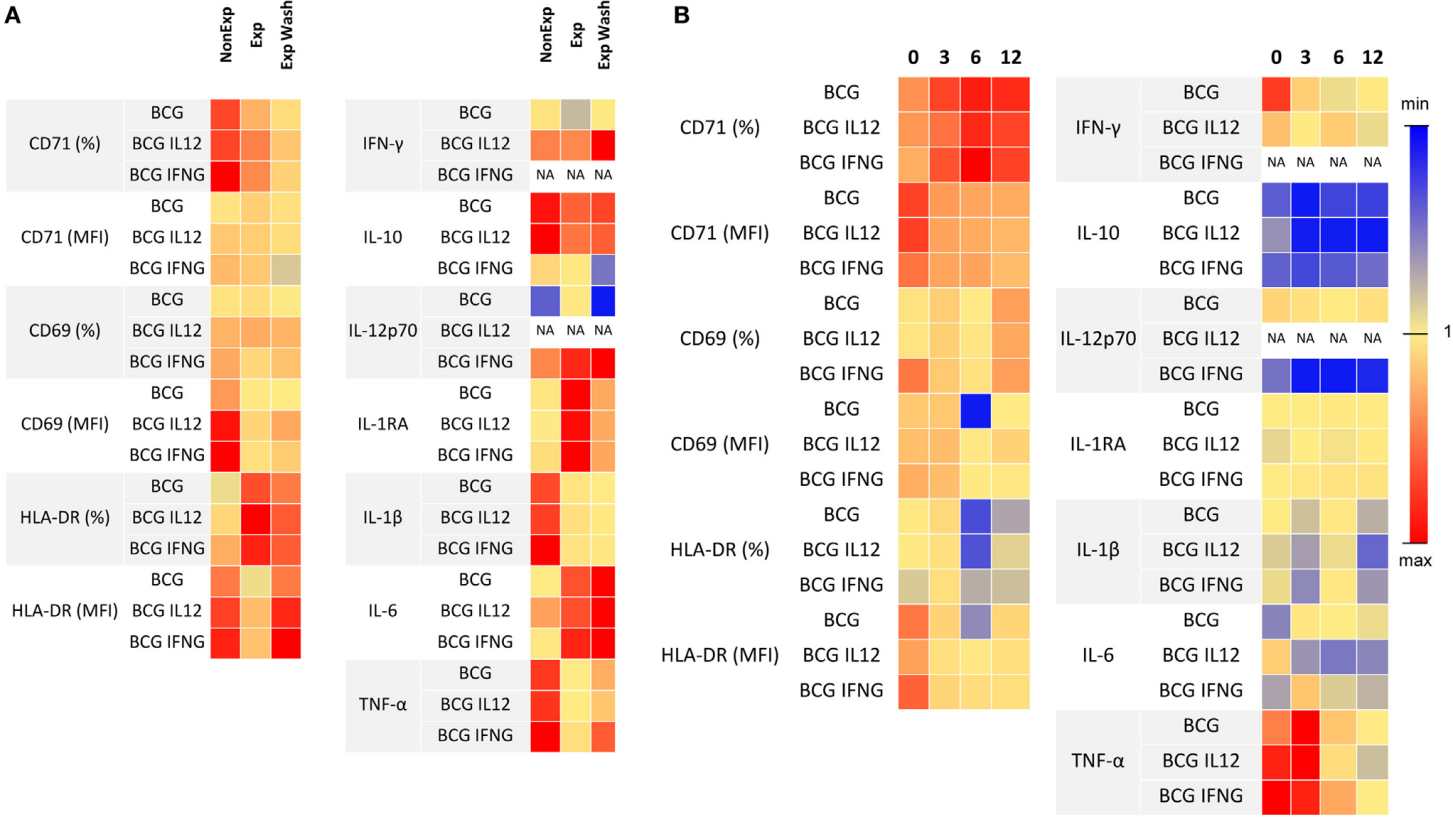
Effects of anti-TNF-α exposure in the IL-12/IFN-γ pathway. **(A)** Stimulation ratio of lymphocyte activation markers expression (measured with flow cytometry) and secreted cytokines (measured with luminex) is represented as a heat map. Stimulation ratio is calculated as “stimulated culture condition/baseline condition.” **(B)** Ratio of lymphocyte activation markers expression and secreted cytokines after Bacillus Calmette–Guérin (BCG) culture using washed blood and non-washed blood during follow-up is represented as a heat map. Blue indicates the minimum values, red the maximum, and yellow next to one for each parameter. Adalimumab-exposed infants, *n* = 4; infliximab-exposed infants, *n* = 2.

Exposure to anti-TNF-α during pregnancy reduced the response after mycobacterial challenge (Figure 5A; Table 3). Exposed-infants presented at birth a lower stimulation ratio (SR, stimulated condition/basal condition) of CD69 (*p* = 0.004) and HLA-DR (*p* = 0.003) MFI (expression per cell). CD69 expression was partially recovered after drug removal (*p* = 0.02) although still reduced (*p* = 0.004); drug removal had a mild effect on HLA-DR SR reduction (*p* = 0.05). There was a tendency toward a reduction of CD71^+^ frequency SR, with (*p* = 0.06) or without (0.06) drug presence.

At birth, TNF-α induction was reduced in the exposed-infant (*p* = 0.0002), being partially recovered after drug removal (*p* = 0.005) although still reduced (*p* = 0.02). Although without statistical significance, IL-1ß secretion was reduced (*p* = 0.06), and IL-6 (*p* = 0.06) and IL-1RA (*p* = 0.07) SR were increased. IL-1ß was not recovered after washing, IL-1RA SR was rescued and IL-6 secretion increased even more (*p* = 0.03). Without stimulation, washed samples from exposed infants produced higher amounts of IL-17 (measured by ELISA) compared with the non-washed condition (*p* = 0.03) and with non-exposed infants (*p* = 0.02). On the other hand, after BCG stimulation, IL-17 production in exposed infants was reduced, as none of the exposed infants produced any detectable IL-17 but one; however, differences did not reach statistical significance. Differences between non-washed and washed samples from exposed infants were maintained (*p* = 0.03) but disappeared when compared with non-exposed infants (*p* = 0.95) (Figure S9 in Supplementary Material). Altogether, these data suggest that immune system activation upon mycobacterial challenge may be compromised.

**Figure 6:**
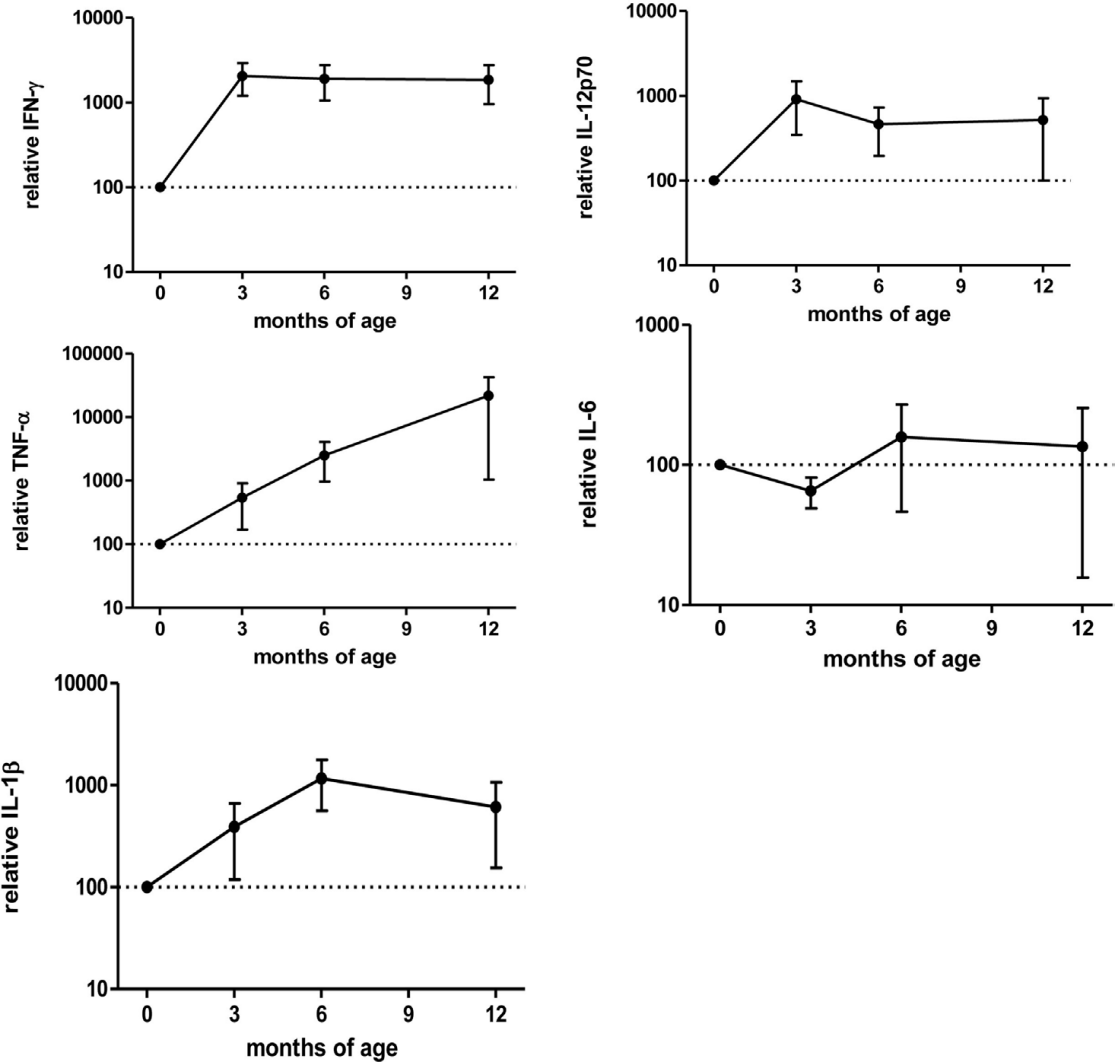
Cytokine secretion in the first months of age related to birth in exposed infants. Cytokine secretion measured by Luminex after 48 h culture with Bacillus Calmette–Guérin. Values are relative to “birth” (0) time point. Points represent mean and bars SE of the mean. Adalimumab-exposed infants, *n* = 3; infliximab-exposed infants, *n* = 1 Tables.

After anti-TNF-α clearing (3–12 months after birth), IFN-γ, IL-12p70, IL-1β, and TNF-α production increased while IL-6 production was stable (Figure 6) and the differences between washed and non-washed blood of exposed infants decreased (overall effect of time on the ratio between washed and non-washed blood, *p* = 0.006). This effect was observed in CD69 (*p* = 0.009) and HLA-DR (*p* = 0.0009) MFI, CD71^+^ frequency (*p* = 0.003), and TNF-α secretion (*p* = 0.02) (Figure 5B).

### Clinical Manifestations

All children showed normal growth and neurological development. Although one child suffered from recurrent infections from 6 to 12 months of age, no other exposed-infants manifested any significant infections, despite normal microbial exposure (attendance to daycare or siblings below 5 years old). Although our recommendation was to avoid rotavirus vaccination, it was used in four exposed-infants with no adverse effects. Atopic dermatitis was observed in four children (two of them without family history), and food allergy was diagnosed in one of them (Table 4).

**Table 4:** Clinical evolution of exposed infants.

ID	Gender	Treatment	Blood follow-up	General information	Family history	Siblings	Infections	Other clinical symptoms	Vaccinations
1	Male	ADA + Pred	No	Normal development, 2w BF, daycare from 9 m	Father with atopic dermatitis and allergic conjunctivitis	Yes. Exposed to ADA and Pred. No significant infections, or atopy	No severe infections. 2 upper respiratory tract infections, 2 gastroenteritis	Atopic dermatitis	Yes, including Prevenar, varicella and MMR

2	Male	ADA + AZA	18 m	Normal development, 3 m BF, daycare from 12 m	Father with psoriasis and allergic rhinitis	NO	No severe infections. 3 gastroenteritis, 3 upper respiratory tract infections, 1 conjunctivitis, 1 bronchitis, and 2 otitis.	At 2y, auto-limited tics	Yes, including varicella, MMR, and RotaTeq^®^ without adverse effects. Urticarial reaction, facial edema, and fever after Prevenar

3	Male	ADA	18 m	Normal development, 5 m BF, daycare from 5 m	Mother with atopic dermatitis and allergic rhinitis	NO	No severe infections. 2 upper respiratory tract infections, 5 recurrent bronchitis, 4 otitis media, and 1 conjunctivitis. Most infections between 6 and 12 months.	Atopic dermatitis and egg allergy	Yes, including *p* Prevenar, varicella, MMR and RotaTeq^®^ without adverse effects.

4	Male	IFX	18 m	Normal development, BF continues at 2y, no daycare	NO	Yes. Exposed to mesalazine. No infections but atopy	No severe infections. 3 bronchitis, thrush that responded to treatment, 1 tonsillitis with otitis media without complications, and 3 upper respiratory tract infections without complications.	Atopic dermatitis	Yes, including Prevenar, varicella, MMR, and RotaTeq^®^ without adverse effects

5	Female	IFX + ADA	12 m	Normal development, no BF, no daycare	NO	NO	No severe infections. 1 otitis media; 1 upper respiratory tract infection complicated with bronchitis; and 2 urinary tract infections without pyelonephritis.	Atopic dermatitis	Yes, including Prevenar and MMR without adverse effects

6	Female	ADA	3 m	Normal development, BF continues at 12 m, daycare from 7 m	NO	NO	No severe infections. 4 otitis media. Mouth-hand-feed infection	NO	Yes, including Prevenar and MMR without adverse effects

7	Male	ADA	12 m	Normal development, no BF, no daycare	NO	Yes, exposed to ADA without significant infections or atopy	No severe infections. 2 upper respiratory tract infections, not complicated	NO	Yes, including Prevenar, MMR and RotaTeq^®^ without adverse effects

*ADA, adalimumab; AZA, azathioprine; IFX, infliximab; Pred, prednisone; w, week; m, month; y, year; BF, breastfeeding*.

## Discussion

In accordance with published data (11, 13), anti-TNF-α mAbs were detectable until 6 months post-partum. Exceptionally, anti-TNF-α mAbs were detectable at 12 months in one exposed-infant. Our results on the child/mother ratio level show some differences from those of a recent publication: mean ratio of 0.99 for ADA and 3.25 for IFX vs reported ratios of 1.21 and 1.97, respectively (13). These differences may be justified because we measured mother’s trough levels during pregnancy instead of levels at birth; also, all patients included received the treatment only 1 week before delivery, while in Julsgaard et al. patients received the last dose from 0 to 25 weeks before delivery. Of interest, we observed a greater ratio with IFX than ADA, attributable to the larger inter-dose interval of IFX than ADA (4–8 folds), and all patients received the last dose a week prior to birth. These discrepancies in the “transferred dosage” of anti-TNF-α may explain why effects observed seemed to be stronger in IFX-exposed infants. Our recommendation would be to try to separate as much as possible from birth the administration of anti-TNF-α in the mother to reduce the drug level in the newborn. In this sense, there are other studies recommending ADA and IFX discontinuation after week 20 of pregnancy to try to reduce drug levels in the newborn (28–30).

In some patients, it has been seen that anti-TNF-α exposure during pregnancy can lead to neutropenia (28). Neutrophils from four children exposed to IFX during pregnancy (including third trimester, without other immunosuppressants) were decreased when measured “a few days” or 15 days after birth, with levels below <0.5 × 10^9^ ANC/L in 3 and 1.1 × 10^9^ ANC/L, and increased 3 months after birth; infectious skin lesions occurred during neutropenia. In our study, three exposed infants had normal levels of neutrophils at birth in cord blood, two had values below the reference range for the age (4.3–11.4 × 10^9^ ANC/L): 2.78 (exposed to ADA + steroids) and 3.06 × 10^9^ ANC/L (exposed to IFX + AZA) and one had values in the limit of the reference range: 4.73 × 10^9^ ANC/L (exposed to ADA + AZA). We observed a decrease of neutrophil levels below the reference ranges at 3 months of age (2.2–6.3 × 10^9^ ANC/L): one infant with severe neutropenia (0.25 × 10^9^ ANC/L; exposed to ADA) and two with moderate neutropenia (1.35 and 1.14 × 10^9^ ANC/L, exposed to ADA + AZA and ADA, respectively). None of them presented infectious skin lesions. Differences in the results may be due to differences in the drug infusion pattern. In the four cases described, the infusion of the last IFX dose was, at least, 8 weeks’ prepartum. Instead, patients included in our study received the last dose of either IFX or ADA from 3 to 7 days’ pre-partum. Also, other factors (such as prematurity and presence of positive neutrophil-specific CD16 autoantibodies) differentiate our cohort from the cases published by GuiddirT et al. However, we agree that neutrophil count should be routinely performed in infants exposed to anti-TNF-α drugs during pregnancy, especially in the event of an infection.

We have observed normal numbers of B cells at birth, although with a more immature phenotype. It is known that TNF-α knock out mice presented abnormal B cell structures. They lack splenic B cell follicles, organized follicular dendritic cell networks and germinal centers. These alterations lead to a decreased humoral response and increased infection risk (20, 21). Nonetheless, mice exposed to anti-TNF-α mAb during gestation did not show any abnormal B cell structures. This difference might be ascribed to the fact that in mice, B cell development occurs 3 weeks after birth. Instead, in humans B cell development occurs during the third trimester of pregnancy and through 8 weeks after delivery (29–31). A study on the impact of the exposure to golimumab during pregnancy in macaques revealed no effect in B or T cell frequency, nor in humoral responses, or in lymphoid organ formation, but maturation status of B cells was not assessed (32). Data from animal models along with our study reinforce the theory that TNF-α plays a role in B cell development and maturation in humans.

Based on empirical experience [adverse event to BCG vaccine (15) and theoretical knowledge (33)], the use of all live vaccines is delayed from 6 to 12 months of age in infants exposed to ADA or IFX during the late second and third trimester of pregnancy (13, 15). Here, we provide objective data to ponder this statement: at birth, exposed infants showed more immature B- and T-cell subsets. However, we observed a normal T-cell proliferation to mitogens, as well as T- and B-cell numbers and maturation, Ig production, and inactivated vaccine responses, accomplishing the criteria for attenuated vaccination in patients with cellular immunodeficiency (34). One infant presented B-cell lymphopenia at birth after ADA + AZA exposure; it is known that AZA exposure during pregnancy can lead to B cell lymphopenia at birth (35). Also, none of the four infants who received rotavirus-inactivated vaccine presented adverse events.

Immune system dysregulation needs to be considered: four of seven of our children presented atopy in the first year (two of them without family history), and all ADA-exposed infants had increased eosinophil counts in cord blood. Exposed infants showed an altered T- and B-regulatory compartment. There was an increased Breg frequency, a population having an anti-inflammatory role in cord blood (36). By contrast, we can speculate that a decreased Treg cell frequency correlating with increased T-cell response to weak stimulus may be a sign of a more responsive immune system, which might be related to the atopy in these patients. However, we cannot rule out the possibility that this may be influenced by the mother’s disease (37). A decrease on Treg cell population has also been observed in infants born to mothers that had received a kidney transplant and were exposed to immunosuppressive drugs during pregnancy. However, in this case, Treg cell numbers were rescued with age (38). As Treg did not increase over time, clinical evolution of exposed infants should be specifically followed-up, with special attention to allergic, inflammatory, and autoimmune events. More studies with larger cohorts are needed to confirm these results.

We have observed a diminished frequency of Treg cells described as CD4^+^CD25^hi^CD127^low^FoxP3^+^ T cells in all exposed infants compared to healthy controls. Interestingly, there are some publications showing an increase in Treg cells in responder patients after anti-TNF-α treatment (39–41). These differences may be explained by the possibility that (1) It is described that the cells that increase in adult are not natural (CD62L^+^) but induced (CD62L^−^) Treg cells (40). As induced Treg cells are differentiated upon an antigenic insult (42), in the umbilical cord blood, the majority of Treg cells would be expected to be natural Treg as they express high levels of CD62L (43) and (2) the effect of anti-TNF-α drugs on the development of induced Tregs in exposed infants is difficult to assess since as early as at 3 months the amount of anti-TNF-a in blood had significantly decreased. It has been noted that the functional capacity of Tregs after anti-TNF-a treatment is increased. Although it would be interesting to study the inhibitory capacity of Treg cells in exposed infants, we cannot test this in samples from our cohort due to limitations related to cell number requirements.

We have shown that drug exposure decreases the response after a mycobacterial challenge at birth, which did not totally recover after drug cleaning. In adults, anti-cytokine biological treatments are thought to cause an immune-deficiency-like phenotype (17). This should also be applied to infants who, besides, intrinsically present a Th2-biased response (44). It has been observed that there is a decreased production of IL-12 but not of IL-6 after anti-TNF-α therapy in adults (45). Also, that there is a decrease in IFN-γ-producing CD8 T cells and in Th1/Th17 subset with an increase in IFN-γ-producing NK cells (46). Results obtained after BCG stimulation do not correspond with those published; we have observed no significant differences in IL-12p70 production in comparison with non-exposed infants and an increased production of IL-6. However, from our results and others (36, 44, 47–50), it would seem that the immune system of patients with inflammatory diseases and neonates show differentiable characteristics. Also, presence of anti-TNF-a mAbs reduced IL-17 production after BCG stimulation that was rescued after whole-blood washing. The advent of biosimilars will broaden the use of biological treatments in developing countries, some of which have endemic tuberculosis or BCG vaccination soon after birth. Until more investigations are performed, BCG vaccination must be absolutely avoided in exposed infants until recovery of antimycobacterial function is verified or at least until 12 months of age. *In vitro* functional studies would be relevant for this purpose.

Although this study has several strengths, including the thorough immune system analysis, it also has some limitations: our cohort of exposed infants is small, and a broader group would probably provide more robust information. Nevertheless, all observations were consistent from sample to sample. Our study control group included infants born to healthy mothers, since no IBD pregnant women with moderate-to-severe disease were without anti-TNF-α treatment; thus, we have not been able to evaluate the effect of IBD itself. Finally, immunological follow-up of healthy controls was not performed for ethical reasons.

This study is the first thorough evaluation of the impact of prenatal anti-TNF-α on the immune system development of exposed-infants. Although we observed specific changes, infants were not clinically compromised. Our results aim at generating consciousness of the need to further study and follow-up on exposed-infants. The pediatrician should be informed of the mother’s mAb treatment during pregnancy, because of the impact on vaccine recommendations, especially with regards to BCG due to the observed mycobacterial-deficient response.

## Ethics Statement

This study was carried out in accordance with the recommendations of Ley General de Sanidad (25/4/1986) Art. 10, with written informed consent from all subjects. The protocol was approved by the ethics committee of the Hospital Sant Joan de Déu (Comité Ético de Investigaciones Clínicas number PIC-50-12). All patients included in the study signed the informed consent, complying with current legislation.

## Author Contributions

AE-S performed immunological studies, carried out the analyses, drafted the initial manuscript, and approved the final manuscript as submitted. IT performed the clinical management of pregnant women, critically reviewed the manuscript, and approved the final manuscript as submitted. AD-M performed clinical follow-up of exposed infants, critically reviewed the manuscript, and approved the final manuscript as submitted. ER and MG performed the clinical management of IBD patients, critically reviewed the manuscript, and approved the final manuscript as submitted. MT, NM, and EG performed anti-TNF-α monitoring, critically reviewed the manuscript, and approved the final manuscript as submitted. JY coordinated anti-TNF-α monitoring, analyzed anti-TNF-α monitoring results, critically reviewed the manuscript, and approved the final manuscript as submitted. AP and MJ conceptualized and designed the study, critically reviewed the manuscript, and approved the final manuscript as submitted. LA performed clinical follow-up of exposed infants, conceptualized and designed the study, critically reviewed the manuscript, and approved the final manuscript as submitted.

## Conflict of Interest Statement

The authors declare that the research was conducted in the absence of any commercial or financial relationships that could be construed as a potential conflict of interest.
