# Supplementary material for: Immunological Changes in Blood of Newborns Exposed to Anti-TNF-α during Pregnancy
Source: Front Immunol. 2017 Sep 21;8:1123. doi: 10.3389/fimmu.2017.01123 (PMC5613099; doi:10.3389/fimmu.2017.01123)
Supplement: Supplementary file 1 [file data_sheet_1.docx]

Supplementary Material

Immunological changes in blood of newborns exposed to anti-TNF-α during pregnancy.

Ana Esteve-Sole, Àngela Deyà, MD PhD, Irene Teixidó MD, Elena Ricart MD PhD, Macarena Gompertz MD, Maria Torradeflot, Noemí de Moner, Europa Azucena Gonzalez, Ana Maria Plaza MD PhD, Jordi Yagüe MD PhD, Manel Juan MD PhD, Laia Alsina MD PhD*

*** Correspondence:**Laia Alsina.

Allergy and Clinical Immunology Department, Hospital Sant Joan de Déu, Institut de Recerca Pediàtrica Hospital Sant Joan de Déu, Esplugues de Llobregat, Spain; Functional Unit of Clinical Immunology Sant Joan de Déu-Hospital Clinic.

lalsina@sjdhospitalbarcelona.org

# Supplementary Data

**Supplementary methods**

**Anti-TNF-a monitoring:**

Levels of anti-TNF-α monoclonal antibody (adalimumab or infliximab) were measured in the serum/plasma of the exposed infants and their mothers with Promonitor ® kits (Movaco, Grifols, Spain) using the Triturus Immunoassay System (Grifols Movaco, Spain). Levels of immunoglobulin A, G, and M were determined by nephelometry (Siemens, Germany).

**Immunophenotyping:**

Immune-phenotype cell staining was performed in heparinized whole blood: for surface staining, 50µL of whole blood was incubated for 15 min at room temperature (RT) with the appropriate monoclonal antibodies (BD Bioscience, San Jose, CA, USA). To lyse erythrocytes and fix cells, stained cells were incubated with 2 ml of BD lysing solution 1x (BD Bioscience) for 15 min at RT. Cells were then washed two times with FACS buffer [phosphate buffered saline (PBS) with 5% fetal calf serum, 0.5% bovine serum albumin, and 0.07% NaN_3_] and samples were acquired using FACSCanto-II (BD Bioscience) cytometer. A minimum of events were acquired for the different populations: 20,000 T cells for T cells subpopulations, 10,000 B cells for B cells subpopulations, and 10,000 lymphocytes for stimulation markers detection. The antibodies used (Ab) are presented in Supplementary Table 2. Gating strategy was previously described (1).

Treg intracellular staining was performed with Treg Detection Kit (CD4/CD25/FoxP3) (Milteny Biotec, Germany) following the manufacturer’s instructions. Briefly, after surface staining performed as described above, cells were fixed with 500 μl of Fixation Buffer for 30 min at 4ºC. Cells were washed two times with FACS buffer and after that incubated with Perm Buffer. To block non-specific Ab binding, cells were incubated with 20 μl of Perm Buffer and 5 μl of FcR Blocking Reagent for 5 min at RT. Cells were then stained with FoxP3-APC Ab for 30 min at 4ºC. Finally, cells were then washed with FACS buffer and acquired by the cytometer.

To study the level of Treg cells in the described reference range, we determined the correlation between the gold standard phenotype for Treg (CD3^+^CD4^+^CD25^hi^CD127^+^FoxP3^+^) and the one in which the reference values are calculated (CD3^+^CD4^+^CD25^hi^CD127^+^). We found a good correlation between the two populations (r: 0.67; p: 0.0009), **Supplementary Figure 6.**

**Intracellular cytokine staining**

For IL-10 production detection, as previously reported (18), cells were maturated 48h with CD40L and LPS and stimulated during the last 5h with LPS, PMA and ionomycin in the presence of Brefaldin A (BFA; 10μl/ml, Sigma-Aldrich, St. Louis, MO, USA). Cells were washed with FACS Buffer and incubated with mAb for anti-human surface molecules for 15 min RT, cells were then permeabilized and then incubated for 30 min, RT, dark with anti-IL-10 Ab. Cells were then washed with FACS buffer and acquired by the cytometer.

**Proliferation analysis**

Peripheral blood mononuclear cells (PBMCs) from hUCB and healthy controls were isolated by Ficoll-Hipaque (Sigma-Aldrich, St. Louis, MO, USA) density gradient centrifugation of heparinized blood. Cells were subsequently washed three times with PBS 1x (Roche Diagnostics, Barcelona, Spain) and cultured with complete medium [RPMI (Gibco, Grand Island, NY, USA) supplemented with 10% heat-inactivated fetal calf serum (FCS; Sigma-Aldrich, St. Louis, MO, USA), 1 μg/ml penicillin, and 1μg/ml streptomycin (Invitrogen, Grand Island, NY, USA)]. Viable cells were counted using a hemocytometer in an inverted microscope.

1.5·10^6^ PBMC/ml was labeled with 5µM 5,6-carboxyfluorescein diacetate succinimidyl ester (CFDA-SE, Invitrogen, Grand Island, NY, USA) for 10 min at 37ºC; then 5mL of cold PBS supplemented with 10% FCS was added for 5 min at 4ºC and washed twice with PBS + 2% FCS before stimulation. CFSE-stained PBMCs (10^6^ PBMCs/ml) were stimulated with phytohemaglutinin A (5µg/mL; PHA, Sigma, St. Louis, MO, USA), pokeweed mitogen (2 µg/mL; PWM; Sigma, St. Louis, MO, USA) and Concavalin A (2 µg/mL; ConA, Sigma, St. Louis, MO, USA) or medium only in a 96-well plate for 7 days at 37ºC in a humidified incubator, with 5% CO2.

We analyzed division index and proliferation index calculated with the FlowJo 7.3 software. Division index is the average number of cell divisions per cell in the original population and it includes the entire population, whereas proliferation index refers to the total number of divisions per proliferating cell as it only takes into account the cells that underwent at least one division(2).

1. Esteve-Solé A, Teixidó I, Deyà-Martínez A, Yagüe J, Plaza-Martín AM, Juan M, et al. Characterization of the highly prevalent regulatory CD24hiCD38hi B-Cell population in human cord blood. Front Immunol [Internet]. Frontiers; 2017 Mar 7 [cited 2017 Mar 7];8:201.

2. Roederer M. Interpretation of cellular proliferation data: Avoid the panglossian. Cytom Part A [Internet]. Wiley Subscription Services, Inc., A Wiley Company; 2011 Feb [cited 2016 Dec 19];79A(2):95–101.

3. Feinberg J, Fieschi C, Doffinger R, Feinberg M, Leclerc T, Boisson-Dupuis S, et al. Bacillus Calmette Guerin triggers the IL-12/IFN-gamma axis by an IRAK-4- and NEMO-dependent, non-cognate interaction between monocytes, NK, and T lymphocytes. Eur J Immunol [Internet]. 2004;34(11):3276–84.
