# Supplementary figures and images for: Immunological Changes in Blood of Newborns Exposed to Anti-TNF-α during Pregnancy
Source: Front Immunol. 2017 Sep 21;8:1123. doi: 10.3389/fimmu.2017.01123 (PMC5613099; doi:10.3389/fimmu.2017.01123)

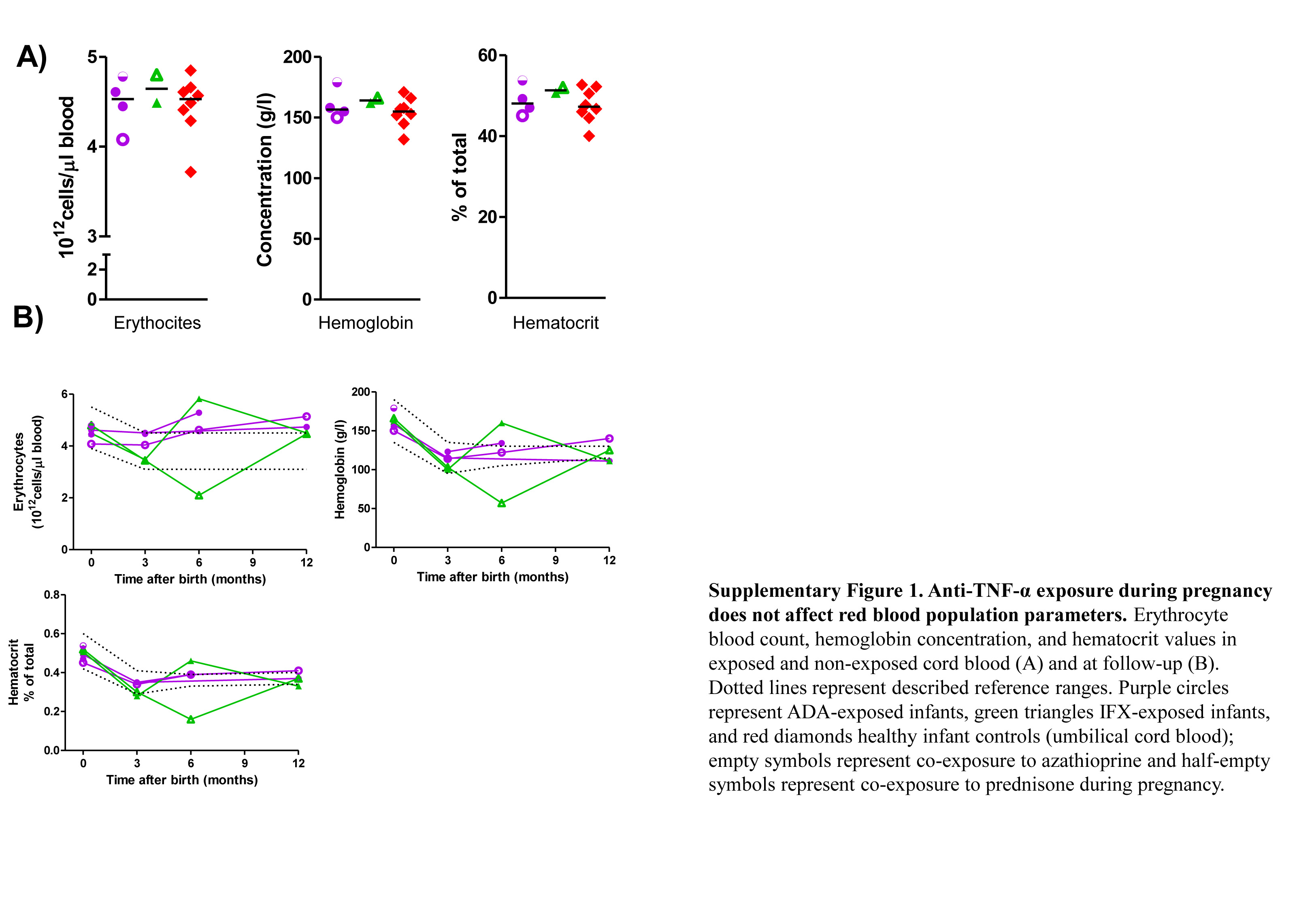

Supplement: Supplementary file 2 [file image_1.jpeg]

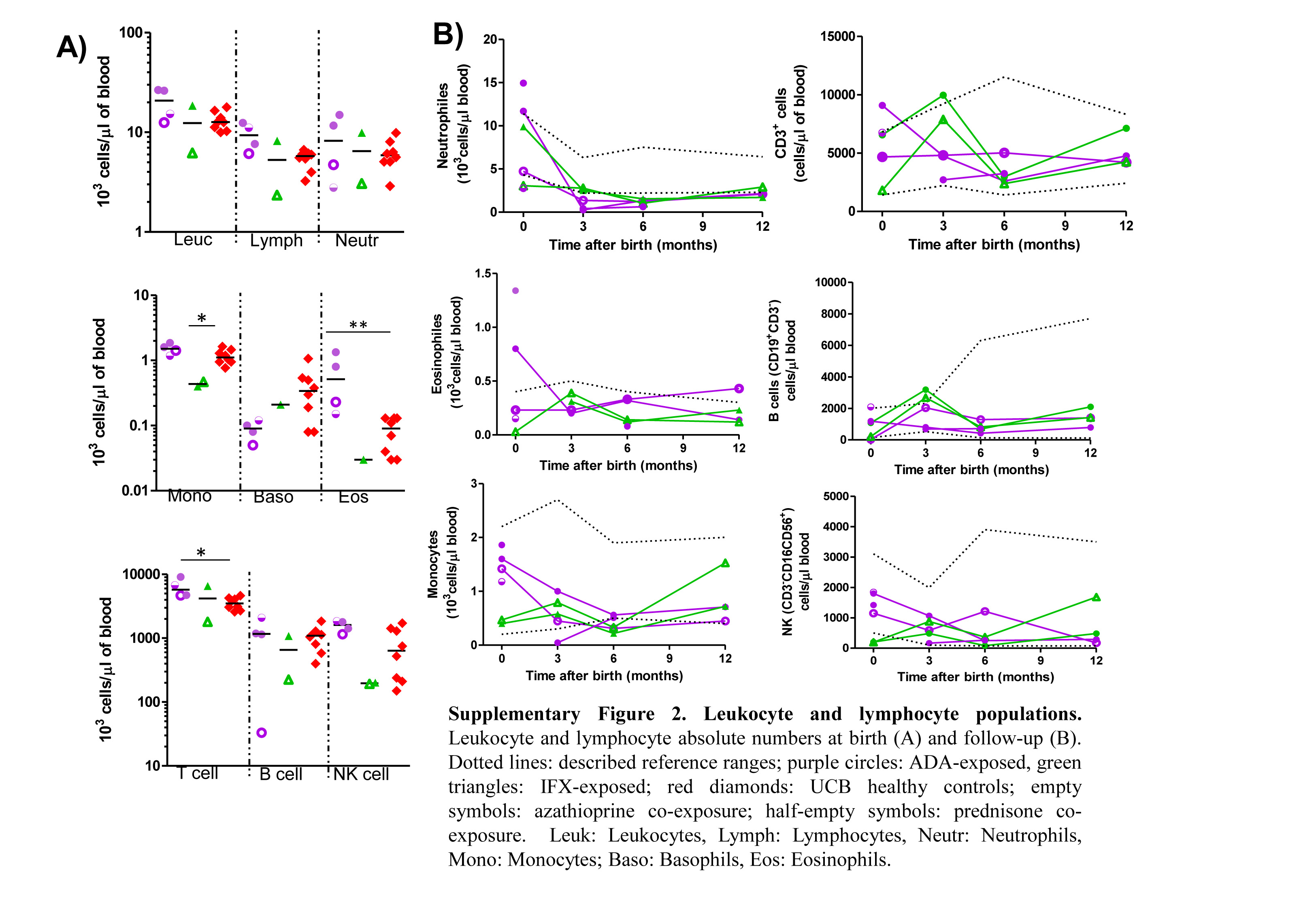

Supplement: Supplementary file 3 [file image_2.jpeg]

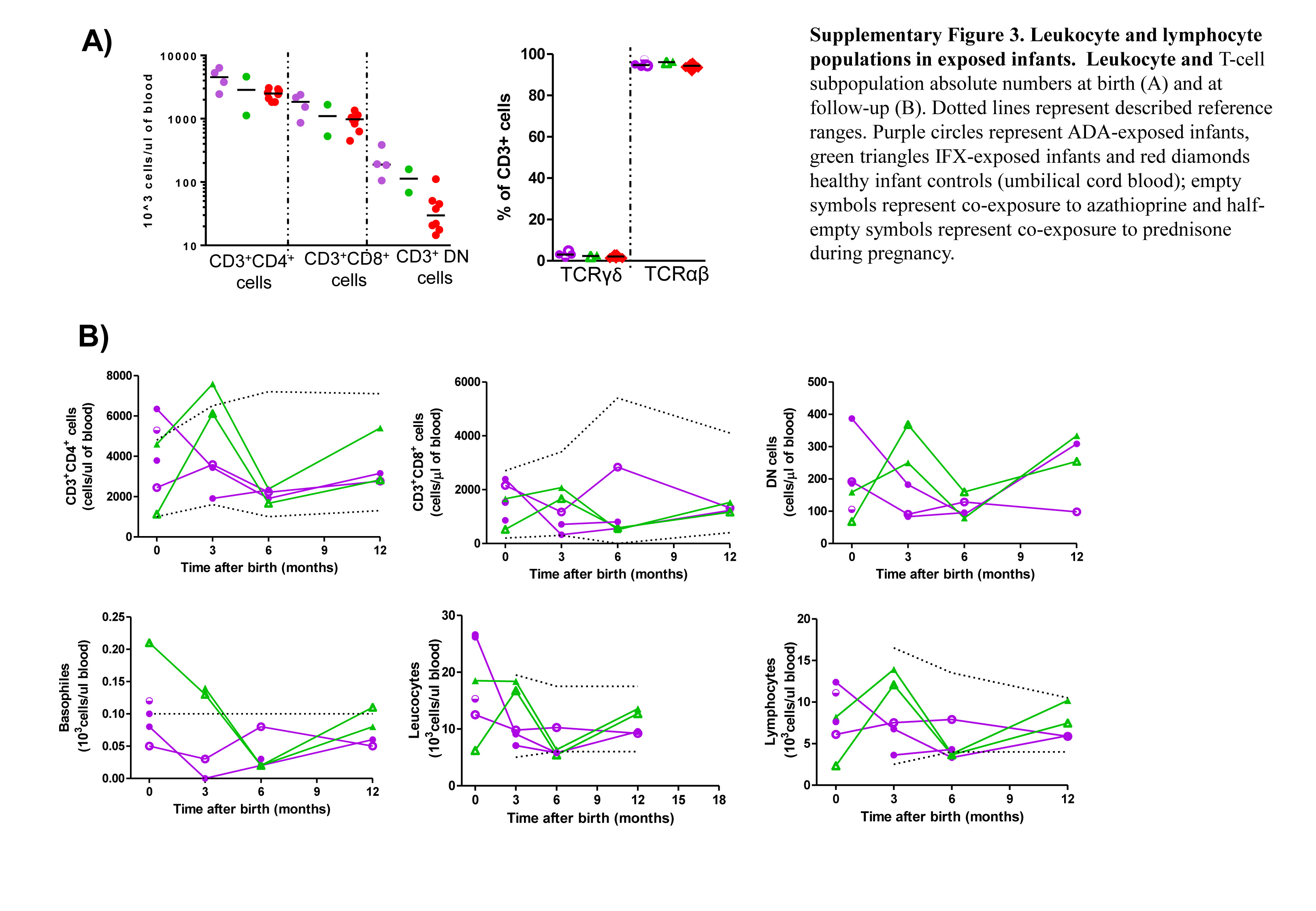

Supplement: Supplementary file 4 [file image_3.jpeg]

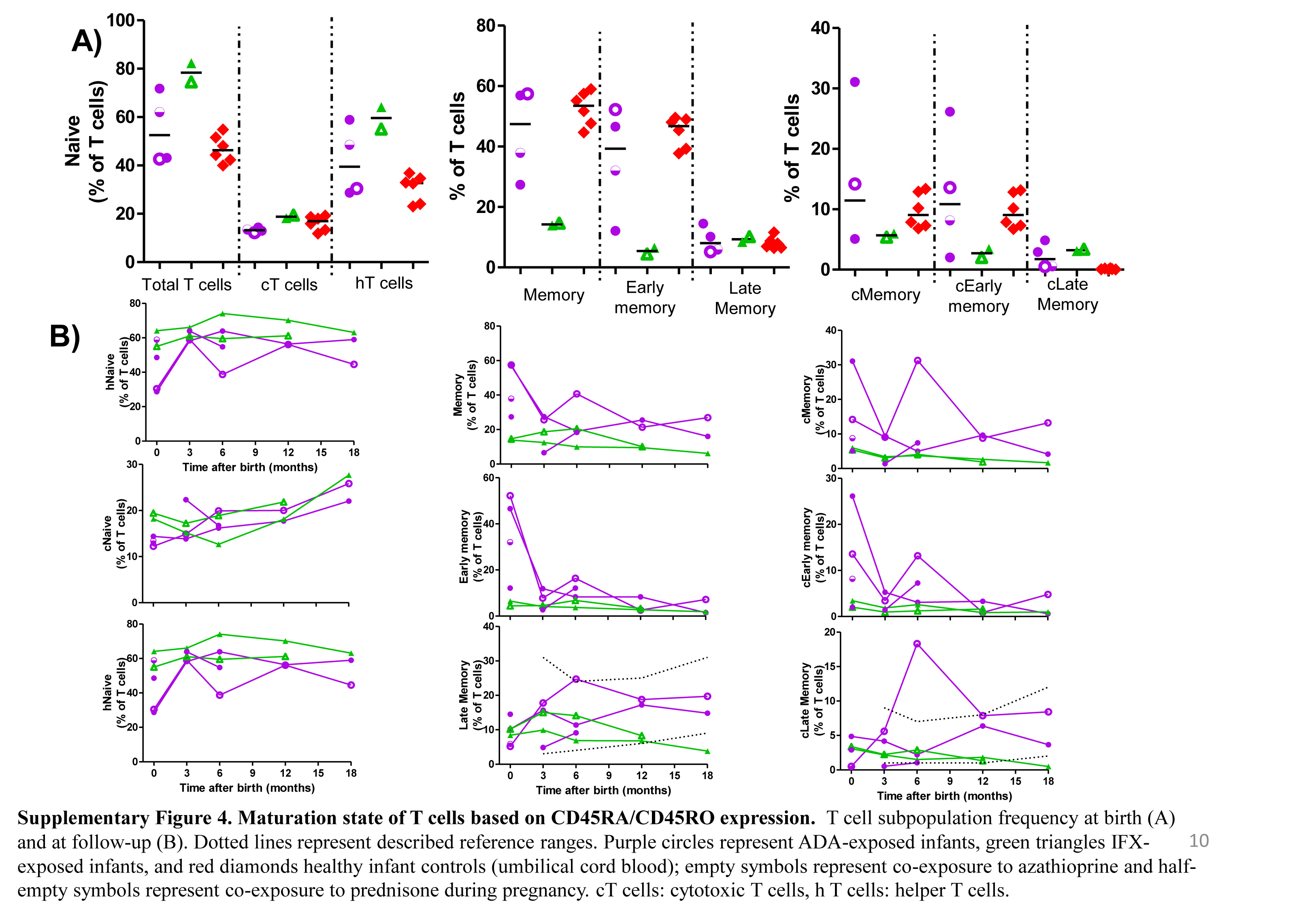

Supplement: Supplementary file 5 [file image_4.jpeg]

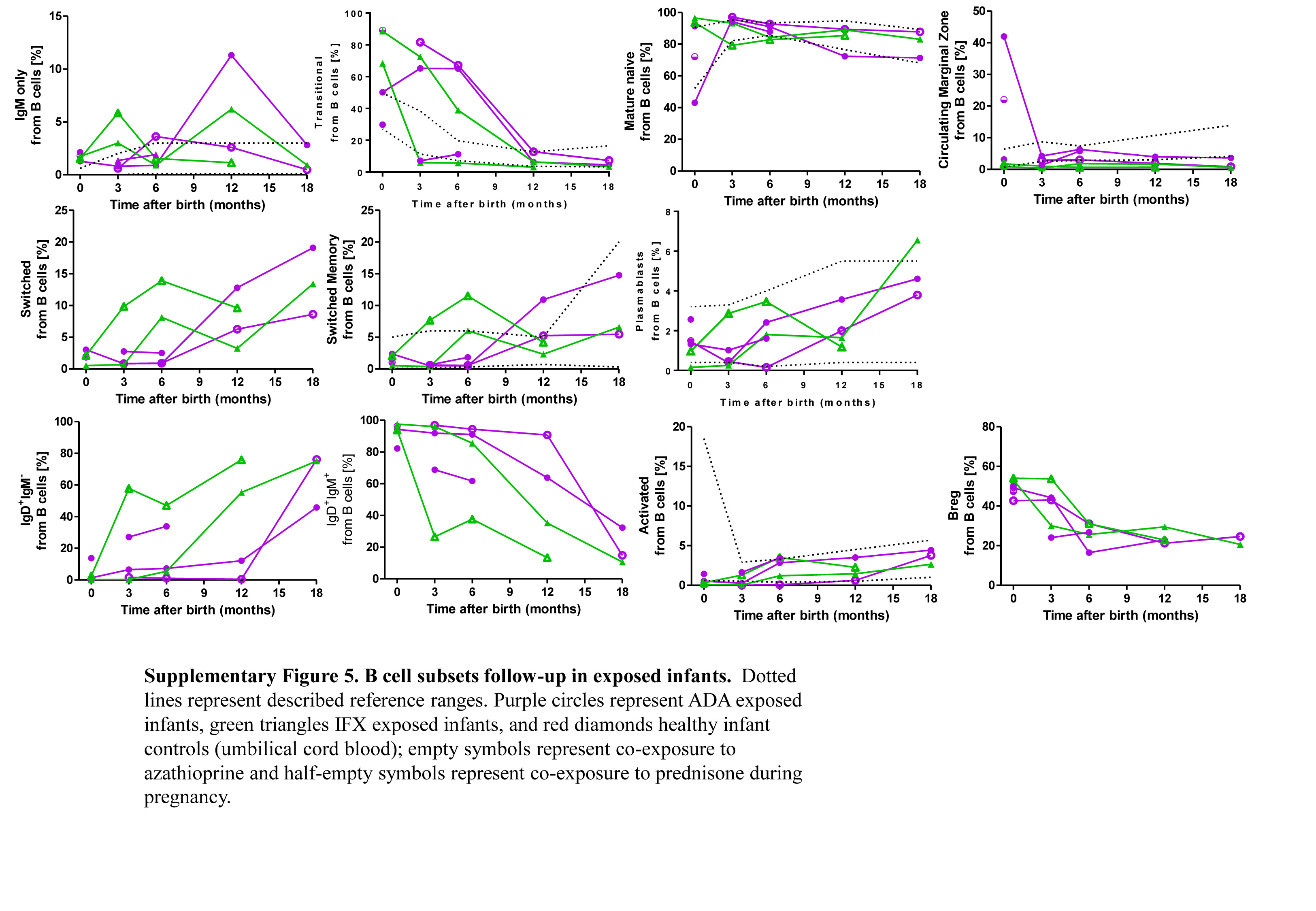

Supplement: Supplementary file 6 [file image_5.jpeg]

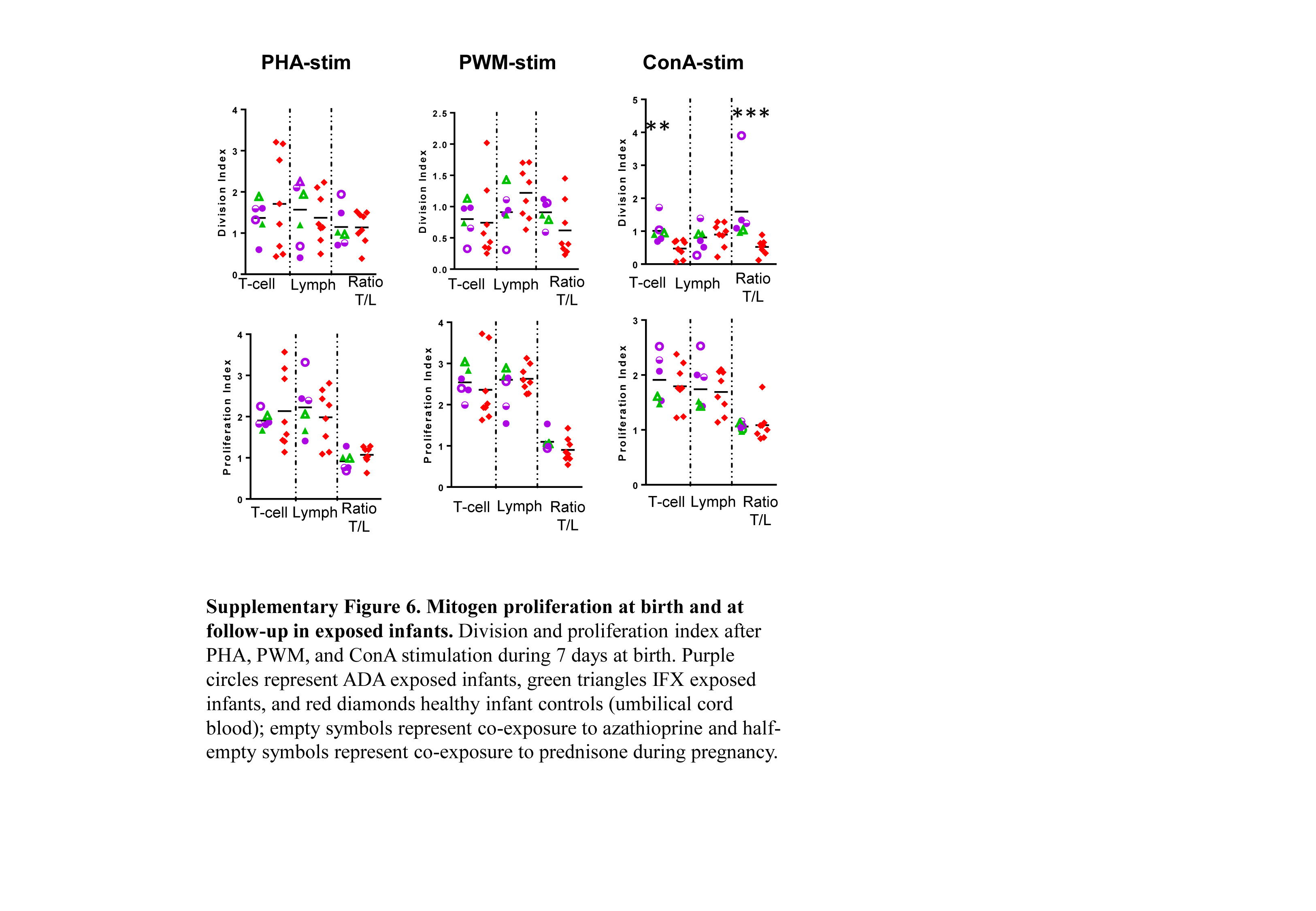

Supplement: Supplementary file 7 [file image_6.jpeg]

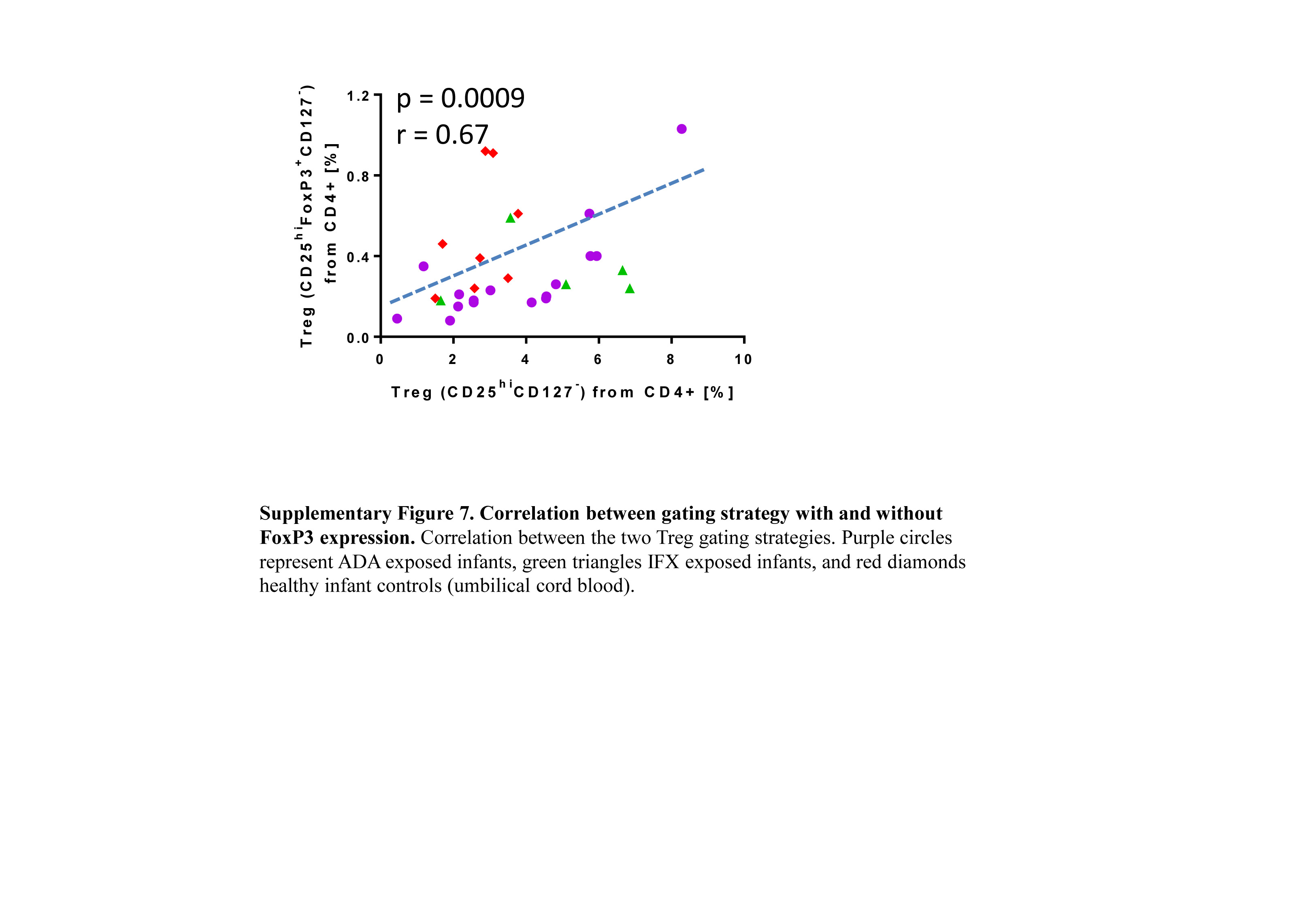

Supplement: Supplementary file 8 [file image_7.jpeg]

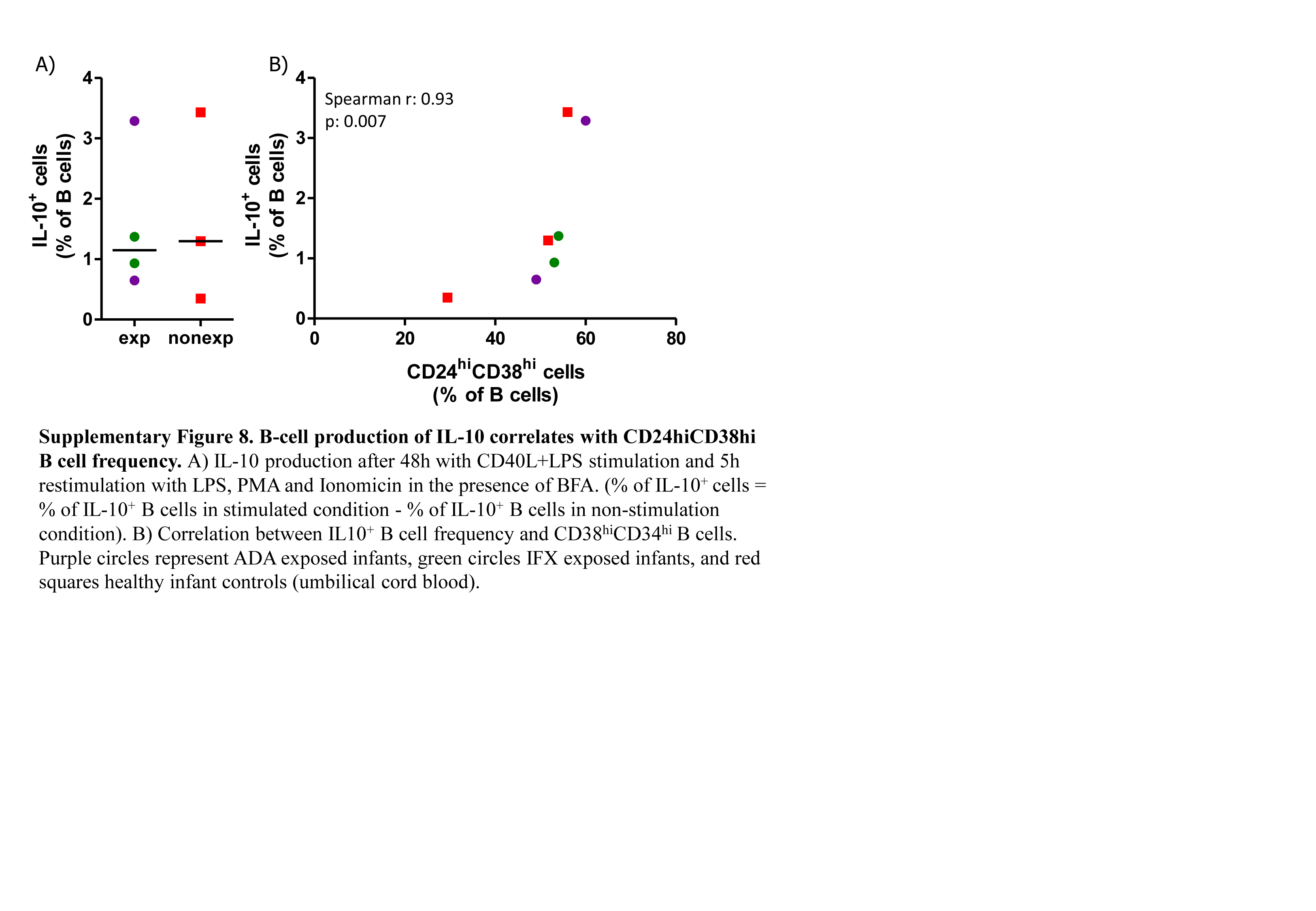

Supplement: Supplementary file 9 [file image_8.jpeg]

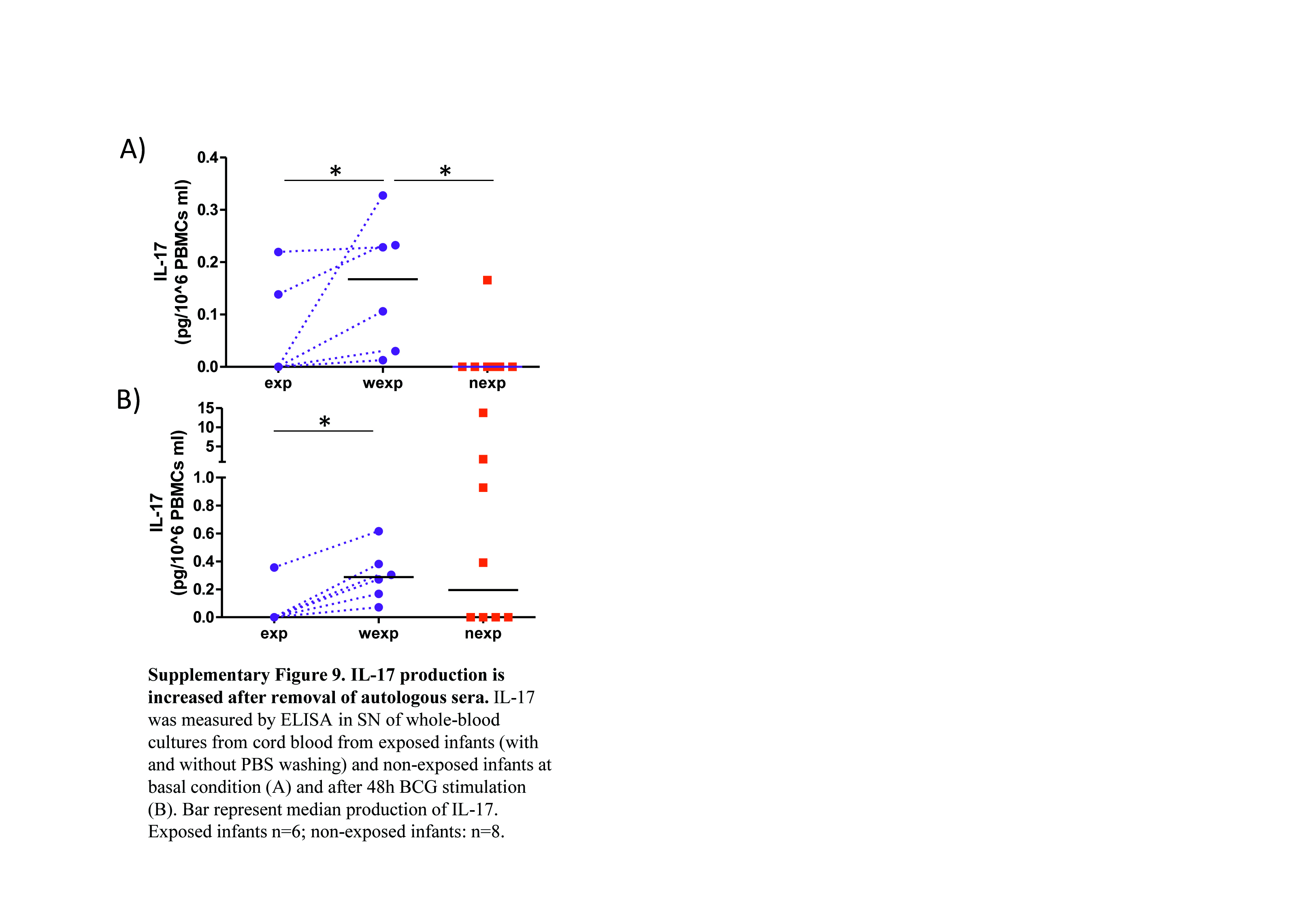

Supplement: Supplementary file 10 [file image_9.jpeg]
