# Supplementary material for: Immunological Changes in Blood of Newborns Exposed to Anti-TNF-α during Pregnancy
Source: Front Immunol. 2017 Sep 21;8:1123. doi: 10.3389/fimmu.2017.01123 (PMC5613099; doi:10.3389/fimmu.2017.01123)
Supplement: Supplementary file 12 [file table_2.docx]

Supplementary Material

Immunological changes in blood of newborns exposed to anti-TNF-α during pregnancy.

Ana Esteve-Sole, Àngela Deyà, MD PhD, Irene Teixidó MD, Elena Ricart MD PhD, Macarena Gompertz MD, Maria Torradeflot, Noemí de Moner, Europa Azucena Gonzalez, Ana Maria Plaza MD PhD, Jordi Yagüe MD PhD, Manel Juan MD PhD, Laia Alsina MD PhD*

*** Correspondence:**Laia Alsina.

Allergy and Clinical Immunology Department, Hospital Sant Joan de Déu, Institut de Recerca Pediàtrica Hospital Sant Joan de Déu, Esplugues de Llobregat, Spain; Functional Unit of Clinical Immunology Sant Joan de Déu-Hospital Clinic.

lalsina@sjdhospitalbarcelona.org

# Supplementary Figures and Tables

**Supplementary Table 2. Antibody panels for umbilical cord and peripheral blood phenotyping.**

| Panel | Antibody - Fluorochrome | | | | | |
| --- | --- | --- | --- | --- | --- | --- |
| T-B-NK phenotype | CD3^1^  APCCy7  SK7 | CD4^2^  FITC  VIT4 | CD8^1^  PerCPCy5.5  RPA-T8 | CD16/CD56^1^  PE  B73/MY31 | CD19^1^  PECy7  HIB19 | CD45^1^  APC  2D1 |
| T-subphenotype | CD3^1^  APCCy7  SK7 | CD8^1^  PerCPCy5.5  RPA-T8 | CD45RA^1^  PECy7  L48 | CD45RO^1^  APC  UCHL1 | TCRab^1^  FITC  WT31 | TCRgd^1^  PE  11F2 |
| B-subphenotype | CD19^1^  PECy7  SJ25C1 | CD21^1^  FITC  B-ly4 | CD38^1^  APC  HIT2 | IgD^1^  PE  IA6-2 | IgM^1^  PerCPCy5.5  SA-DA4 | CD27^3^  PECy7  1A4CD27 |
| T regulatory cells | CD3^3^  AlexaFluor750  UCHT1 | CD4^2^  FITC  VIT4 | CD25^2^  PE  4E3 | CD127^1^  PECy7  HIL-7R-M221 |  |  |
| B regulatory cells | CD19^1^  PECy7  SJ25C1 | CD24^1^  PerCPCy5.5  ML5 | CD38^1^  APC  HIT2 |  |  |  |
| Activation markers | CD11b^1^  PECy7  ICRF44 | CD69^1^  APCCy7  FN50 | CD71^1^  APC  M-A712 | HLA-DR^1^  PerCPCy5.5  G46-6 |  |  |

^1^BD Biosciences; ^2^Milteny Biotech; ^3^Beckman coulter (Brea, CA, USA);
