# Supplementary material for: Immunological Changes in Blood of Newborns Exposed to Anti-TNF-α during Pregnancy
Source: Front Immunol. 2017 Sep 21;8:1123. doi: 10.3389/fimmu.2017.01123 (PMC5613099; doi:10.3389/fimmu.2017.01123)
Supplement: Supplementary file 13 [file table_3.docx]

Supplementary Material

Immunological changes in blood of newborns exposed to anti-TNF-α during pregnancy.

Ana Esteve-Sole, Àngela Deyà, MD PhD, Irene Teixidó MD, Elena Ricart MD PhD, Macarena Gompertz MD, Maria Torradeflot, Noemí de Moner, Europa Azucena Gonzalez, Ana Maria Plaza MD PhD, Jordi Yagüe MD PhD, Manel Juan MD PhD, Laia Alsina MD PhD*

*** Correspondence:**Laia Alsina.

Allergy and Clinical Immunology Department, Hospital Sant Joan de Déu, Institut de Recerca Pediàtrica Hospital Sant Joan de Déu, Esplugues de Llobregat, Spain; Functional Unit of Clinical Immunology Sant Joan de Déu-Hospital Clinic.

lalsina@sjdhospitalbarcelona.org

# Supplementary Figures and Tables

**Supp Table 3. Mother’s clinical data, treatment details, and delivery conditions.**

| **Clinical data** | |
| --- | --- |
| **Age** | 34 (range 27-36y) |
| **IBD** | 6 Crohn  1 ulcerative colitis |
| **IBD duration (years)** | 9 (range 2.5-11 years) |
| **Severity of disease** | Crohn: 4 ileal (3 resections); 2 colonic localizations  Extensive ulcerative colitis |
| **Active disease during pregnancy** | 5 remissions  2 activity (1-3 Tm and 1^st^ Tm) |
| **Other co-morbidities** | 1 epilepsy (treated with levetiracetam + CMZ) + erythema nodosum  1 hypothyroidism |
| **Treatment details** | |
| **Anti-TNF-a dosage during pregnancy** | 5 Adalimumab:  40 mg/2 weeks (4)  40 mg/1 weeks (1)  2 Infliximab  5 mg/kg/8 weeks  10 mg/kg/6-8 weeks |
| **>6 month anti-TNF-a before delivery?** | 7/7 |
| **Anti-TNF-a levels during pregnancy** | Measured 12 days before delivery (4-30 days)  Adalimumab: 4/5: > 4µg/ml (5.2 - >12 µg/ml)  Infliximab: 2/2: > 3 µg/ml (4.1 to 12.4 µg/ml) |
| **anti-TNF-a mAb** | 7/7 negative |
| **Other immunosuppressive treatment during pregnancy** | 3/7  Prednisone 20-40 mg/day (1)  Azathioprine 125-150 mg/day (2) |
| **Pregnancy and labor** | |
| **Other risk factors** | 1 smoker |
| **Pregnancy complications** | 2 mild pre-eclampsia |
| **Delivery** | 4 normal  3 caesarean sections (1 urgent, 2 elective due to previous caesarian section) |
| **Gestational age** | 1 premature 35 weeks (IBD maternal activity)  6 on term: 39 weeks (37-41 weeks) |
| **Apgar 1/5** | 9/10 all |
| **Low weight for gestational age or malformations** | No |
| **Post-partum IBD reactivation** | 2/7 |
