# Supplementary material for: Immunological Changes in Blood of Newborns Exposed to Anti-TNF-α during Pregnancy
Source: Front Immunol. 2017 Sep 21;8:1123. doi: 10.3389/fimmu.2017.01123 (PMC5613099; doi:10.3389/fimmu.2017.01123)
Supplement: Supplementary file 14 [file table_4.docx]

Supplementary Material

Immunological changes in blood of newborns exposed to anti-TNF-α during pregnancy.

Ana Esteve-Sole, Àngela Deyà, MD PhD, Irene Teixidó MD, Elena Ricart MD PhD, Macarena Gompertz MD, Maria Torradeflot, Noemí de Moner, Europa Azucena Gonzalez, Ana Maria Plaza MD PhD, Jordi Yagüe MD PhD, Manel Juan MD PhD, Laia Alsina MD PhD*

*** Correspondence:**Laia Alsina.

Allergy and Clinical Immunology Department, Hospital Sant Joan de Déu, Institut de Recerca Pediàtrica Hospital Sant Joan de Déu, Esplugues de Llobregat, Spain; Functional Unit of Clinical Immunology Sant Joan de Déu-Hospital Clinic.

lalsina@sjdhospitalbarcelona.org

# Supplementary Figures and Tables

**Supplementary Table 4. Anti-TNF-α drug levels.** ADA: adalimumab, IFX: infliximab

|  | **Drug** | **Trough drug level (µg/ml) Mother** | **Drug level (µg/ml)**  **Cord blood** | **Days from last mother’s anti-TNF-α dose to labor** | **Months with detectable drug in blood** |
| --- | --- | --- | --- | --- | --- |
| **1** | ADA | -- | 6.46 | -- | -- |
| **2** | ADA | >12 | 11.48 | 7 | 12 |
| **3** | ADA | >12 | 13.168 | 5 | 6 |
| **6** | ADA | 7.8 | -- | 7 | -- |
| **7** | ADA | 5,2 | 5.87 | 3  0 | -- |
| **4** | IFX | 4.1 | 11.11 | 7 | 6 |
| **5** | IFX | 12.4 | 42.52 | 4  0 | 6 |
